# Evaluating the Performance of Malaria Genetics for Inferring Changes in Transmission Intensity Using Transmission Modeling

**DOI:** 10.1093/molbev/msaa225

**Published:** 2020-09-08

**Authors:** Oliver J Watson, Lucy C Okell, Joel Hellewell, Hannah C Slater, H Juliette T Unwin, Irene Omedo, Philip Bejon, Robert W Snow, Abdisalan M Noor, Kirk Rockett, Christina Hubbart, Joaniter I Nankabirwa, Bryan Greenhouse, Hsiao-Han Chang, Azra C Ghani, Robert Verity

**Affiliations:** 1 MRC Centre for Global Infectious Disease Analysis, Department of Infectious Disease Epidemiology, Imperial College London, London, United Kingdom; 2 KEMRI-Wellcome Trust Research Programme, Centre for Geographic Medicine Research-Coast, Kilifi, Kenya; 3 Population Health Unit, Kenya Medical Research Institute—Wellcome Trust Research Programme, Nairobi, Kenya; 4 Centre for Tropical Medicine and Global Health, Nuffield Department of Clinical Medicine, University of Oxford, Oxford, United Kingdom; 5 Global Malaria Programme, World Health Organization; 6 Wellcome Centre for Human Genetics, University of Oxford, Oxford, United Kingdom; 7 Infectious Diseases Research Collaboration, Kampala, Uganda; 8 Makerere University College of Health Sciences, Kampala, Uganda; 9 Department of Medicine, University of California, San Francisco, San Francisco, CA; 10 Center for Communicable Disease Dynamics, Harvard TH Chan School of Public Health, Boston, MA

**Keywords:** malaria, genetics, surveillance, modeling

## Abstract

Substantial progress has been made globally to control malaria, however there is a growing need for innovative new tools to ensure continued progress. One approach is to harness genetic sequencing and accompanying methodological approaches as have been used in the control of other infectious diseases. However, to utilize these methodologies for malaria, we first need to extend the methods to capture the complex interactions between parasites, human and vector hosts, and environment, which all impact the level of genetic diversity and relatedness of malaria parasites. We develop an individual-based transmission model to simulate malaria parasite genetics parameterized using estimated relationships between complexity of infection and age from five regions in Uganda and Kenya. We predict that cotransmission and superinfection contribute equally to within-host parasite genetic diversity at 11.5% PCR prevalence, above which superinfections dominate. Finally, we characterize the predictive power of six metrics of parasite genetics for detecting changes in transmission intensity, before grouping them in an ensemble statistical model. The model predicted malaria prevalence with a mean absolute error of 0.055. Different assumptions about the availability of sample metadata were considered, with the most accurate predictions of malaria prevalence made when the clinical status and age of sampled individuals is known. Parasite genetics may provide a novel surveillance tool for estimating the prevalence of malaria in areas in which prevalence surveys are not feasible. However, the findings presented here reinforce the need for patient metadata to be recorded and made available within all future attempts to use parasite genetics for surveillance.

## Introduction

Molecular tools are increasingly being used to understand the transmission histories and phylogenies of infectious pathogens (Hall et al. 2015). Using phylodynamic methods, it is now possible to estimate the historic prevalence of infection directly from molecular data, even in organisms with relatively complex lifecycles (Volz et al. 2009). However, these tools typically rely on pathogens having an elevated mutation rate and not undergoing sexual recombination, which allows for the application of coalescent theory (Grenfell et al. 2004). Consequently, these techniques are yet to be adapted for the study of *Plasmodium falciparum* malaria, which is known to undergo frequent sexual recombination. In addition, malaria transmission between both the human and the mosquito hosts involves a series of population bottlenecks (Vaughan 2007; Churcher et al. 2010), which combined with the brief sexual stage involving a single two-step meiotic division (Bennink et al. 2016), have marked effects on the population genetics of *P. falciparum* (McKenzie et al. 2001; Chang et al. 2013). This is extenuated by evidence of cotransmission (multiple parasite strains introduced within an infection event) of clonally related parasites (Wong et al. 2017). This phenomenon, in combination with host-mediated immune (Barry et al. 2007; Portugal et al. 2011) and density-dependent regulation of superinfection (infection of an already infected individual) (Bruce et al. 2000; Pinkevych et al. 2013), results in a complicated network of processes driving the genetic diversity of the parasite population within an individual host.

Despite this substantial complexity, an increasingly nuanced understanding of the processes shaping parasite genetic diversity is appearing, with multiple genetic metrics proving promising for inferring transmission intensity (Daniels et al. 2013; Nkhoma et al. 2013). For example, measures of the multiplicity of *P. falciparum* infections have been shown to be useful for identifying hotspots of malaria transmission (Bejon et al. 2010; Karl et al. 2016). The spatial connectivity of parasite populations has also been shown to be well predicted by pairwise measures of identity-by-descent (IBD) (Omedo, Mogeni, Bousema, et al. 2017; Taylor et al. 2017). More recently, it has been shown that malaria genotyping could be used to enhance epidemiological surveillance (Daniels et al. 2015), however, two main challenges have been identified before molecular tools could be used in an operational context. The first is that our understanding of the relationship between transmission intensity and within-host parasite genetic diversity is incomplete. Combined models of both population genetics and malaria epidemiology would allow us to develop a more detailed view of both processes, yet these two approaches are largely explored separately. Recent efforts have been made to incorporate both modeling scales within one framework (Nguyen et al. 2015), with the concomitant modeling of resistance evolution both within and between hosts yielding important insights into the evolution of drug resistance (Legros and Bonhoeffer 2016). However, the realism of either the transmission process or the genetic evolutionary process has been limited in these models, with the representation of recombination and the parasite lifecycle within the mosquito often simplified. This makes the generalizability of using molecular tools for surveillance difficult. More realistic models are subsequently needed that capture both processes. These models could answer previous hypotheses (Wong et al. 2018) about how transmission intensity alters the rate at which superinfection events and cotransmission of genetically related parasites shape the parasite genetic diversity observed within humans. The second challenge is to understand in what situations molecular tools will offer advantages over traditional surveillance. In addition, power calculations need to be carried out to understand how many samples are required for reliable inference and what types of genetic data are most informative.

Here, we use mathematical transmission modeling to address these challenges. We extend a previously published malaria transmission model (Griffin et al. 2016), which now allows parasite populations to be followed explicitly through the parasite’s obligate sexual life cycle by the inclusion of individually modeled mosquitoes. The new model is fitted to parasite single-nucleotide polymorphism (SNP) genotype data to capture the observed relationship between an individual’s age and their complexity of infection (COI), defined as the total number of genetically distinct parasite strains in an individual. Using the fitted model, we characterize how six measures of parasite genetic diversity respond to changes in transmission intensity. We continue by conducting a power analysis, assessing the ability of each metric to detect changes in transmission intensity as a function of the number of available samples. We conclude by building an ensemble statistical model, which demonstrates how routinely collected clinical genotype samples could be used for accurate prediction of malaria prevalence using as few as 200 SNP-genotyped samples.

## Results

### Complexity of Infection Data

**Fig. 1.**
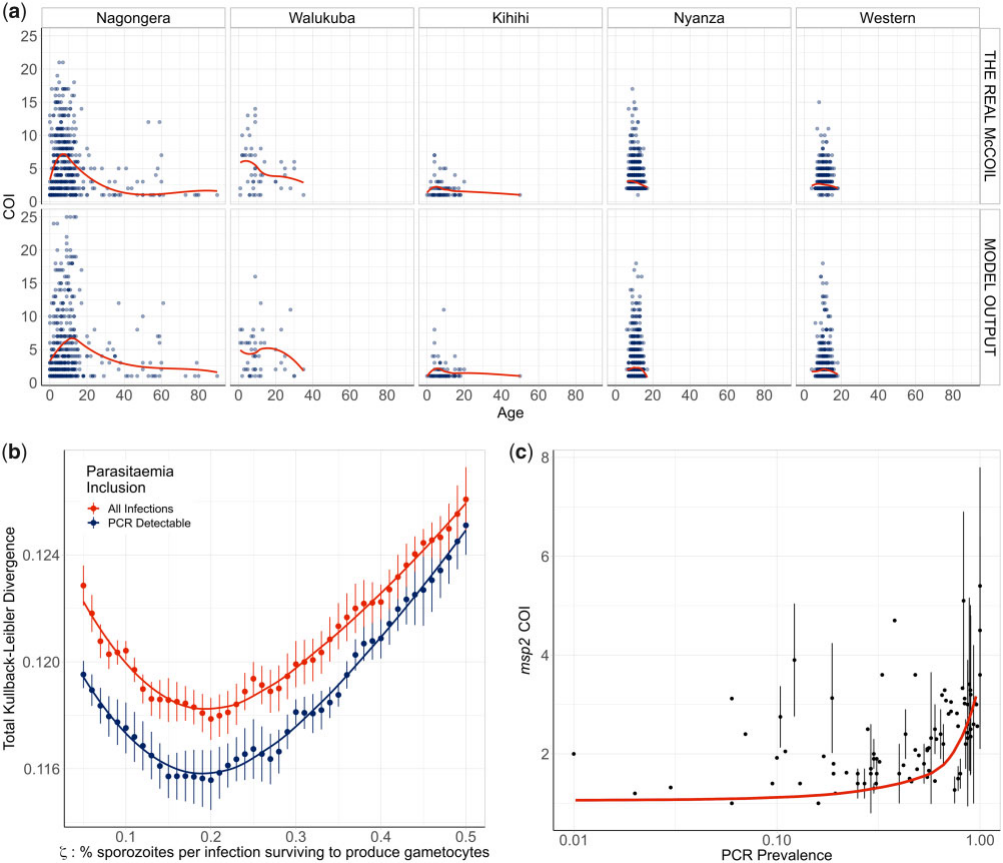
Modeled estimates of the relationship between complexity of infection against age. (*a*) One realization of the model-predicted relationship between complexity of infection (COI) and age compared with the observed relationship estimated using *THE REAL McCOIL*. Each point represents an individual, with a local regression fit plotted in red. The relationship shown represents the selected best model fit, which estimates that 20% of sporozoites successfully progress to blood-stage infection in an individual with no immunity. In (*b*), the results of the model fit are shown, with each point representing the mean Kullback–Leibler divergence and the whiskers representing the 95% confidence interval. Results of model fitting are shown for the assumption that all infections are detected (red) or only those that are PCR-detectable (blue). In (*c*), the model-predicted relationship between COI measured by *msp2* genotyping and PCR prevalence is shown in red, with the point-ranges showing observed values of COI by *msp2* genotyping from the literature review.

First, we used *THE REAL McCOIL* (Chang et al. 2017) to estimate the COI from SNP-genotyped samples collected previously from individuals with evidence of asexual parasitaemia by microscopy from regions in Kenya and Uganda (fig. 1). These two data sets were selected as they recorded both the age of the sampled individuals and the SNP intensities at sufficiently large number of loci, enabling the relationship between COI and age to be estimated. After excluding SNP loci with >20% missing data and subsequently removing samples with >25% missing SNP data from further analysis, the COI was estimated for 2,419 samples from 95 primary schools in Western Kenya (1,363 from Nyanza province and 1,056 from Western province) and 584 samples from representative cross-sectional household surveys in three subcounties in Uganda (462 from Nagongera in Tororo District, 74 from Kihihi in Kanungu District, and 48 from Walukuba in Jinja District) (see table 1). Distribution of COI varied between each region, ranging between 1 and 21 and broadly peaking in children aged 6 years old before decreasing with increasing age of the individual sampled.

**Table 1.** Study Site Age and Sample Size.

	Kenya		Uganda		
	Western Kenya	Nyanza	Nagongera, Tororo District	Kihihi, Kanungu District	Walukuba, Jinja District
Samples	1,363	1,056	462	74	48
Mean age, years (range)	11.1 (4–18)	11.0 (6–17)	10.9 (0–90)	9.0 (0–50)	11.2 (1–35)
Reference	Omedo, Mogeni, Rockett, et al. (2017). Wellcome Open Res. 2:1–25		Chang et al. (2017). PLoS Comput Biol. 13:e1005348		

### Model Fitting

We developed an extended version of a previously published individual-based model of malaria transmission (Griffin et al. 2016). Briefly, the model was extended to include individual mosquitoes, enabling parasite populations and their genotypes to be tracked throughout the full lifecycle, enabling the potential formation of multiple oocysts from an infectious event and multiple genetically distinct sporozoites to be onwardly transmitted. Male and female gametocytes are sampled from the infecting human with the probability proportional to relative densities of each genotype. The resultant oocyst is able to produce up to four new parasite genotypes resulting from a two-step meiotic division. The extensions require to define the proportion of sporozoites from an infectious bite that survive to found a blood-stage infection, which we define as *ζ*. This process will ultimately affect the level of new parasite genetic diversity introduced and consequently we parameterized our developed model (see supplementary methods, Supplementary Material online) through fitting to the earlier estimated relationships between COI and age in the five regions across Uganda and Kenya (fig. 1*a*). We estimate that 20% of sporozoites onwardly transmitted within an infectious bite successfully progress to a blood-stage infection and produce gametocytes that may contribute to future mosquito infections. The model captures the observed peak in COI observed at age 7–8 years (fig. 1*a*); however, the comparatively fewer samples at higher ages make it difficult to confirm that this is the true peak in COI (see supplementary table 1, Supplementary Material online). Additionally, this observed peak in COI also likely reflects the limits of detection, with more accurate model predictions occurring under the assumption that parasite strains that would not be detected by PCR do not contribute to the estimated COI (fig. 1*b*). Model fitting also showed that sensitivity of the model fit to the percentage of sporozoites that survive is negligible between values of 15–20%, with the confidence intervals for the most likely parameter value of *ζ* overlapping intervals for values of *ζ* ranging from 0.1 to 0.29.

To further assess the fitted model, we wanted to incorporate estimates of COI based on *msp2* genotyping, which is more commonly measured, however, it does underestimate COI in individuals with high COI, with COIs >7 difficult to resolve. We updated a previous literature review (Karl et al. 2016) of paired estimates of *msp2* COI and parasite prevalence by PCR, which yielded 91 paired measures of *msp2* COI and PCR prevalence. The fitted model predicts an increase in *msp2* COI with increasing malaria prevalence in agreement with the data collected within our literature search (fig. 1*c*). However, there are notably larger uncertainties in the recorded *msp2* COI at higher prevalence ranges in the studies found.

### Contribution of Cotransmission Events to Within-Host Parasite Diversity

**Fig. 2.**
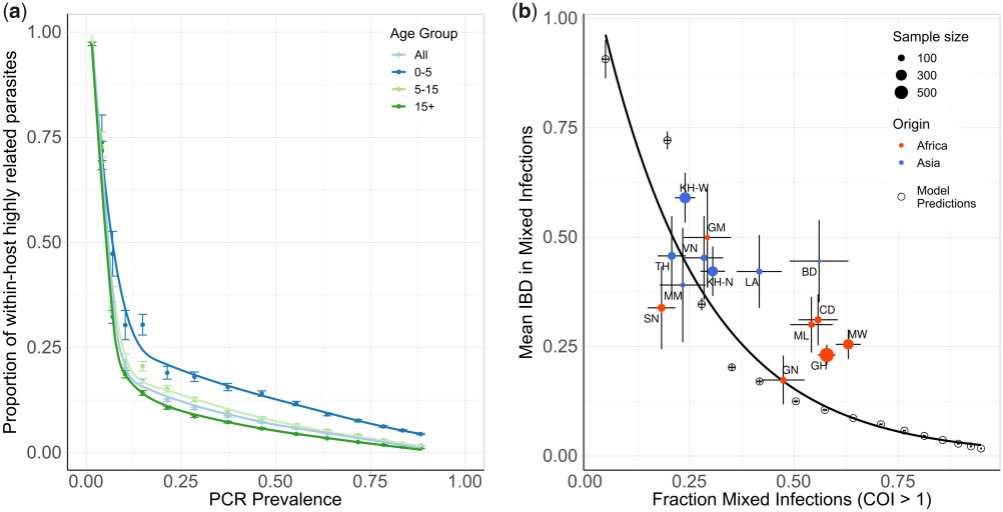
Contribution of superinfection and cotransmission to within-host parasite relatedness. In (*a*), the model-predicted relationship between the mean within-host proportion of highly identical parasite strains (>50% of loci comparisons are identical by descent [IBD]) against PCR prevalence. The relationship is shown for all ages and for three age groups: 0–5 years, 5–15 years, and 15+ years, with error bars showing ±1 SEM. In (*b*), the mean IBD in mixed infections (COI>1) is shown against the proportion of mixed infections. Results from model simulations are shown with empty circles with an exponential regression shown with the black curve. The model estimates are compared with estimates of IBD from whole-genome sequence data collected in sites across Africa and Asia, which were estimated previously in Zhu et al. (2019). Populations are colored by continent, with size reflecting sample size and error bars showing ±1 SEM. Abbreviations: SN, Senegal; GM, The Gambia; NG, Nigeria; GN, Guinea; CD, The Democratic Republic of Congo; ML, Mali; GH, Ghana; MW, Malawi; MM, Myanmar; TH, Thailand; VN, Vietnam; KH, Cambodia; LA, Laos; BD, Bangladesh.

Using the fitted model, we explored the relationship between the proportion of within-host parasite strains that are highly related, which we define as being >50% IBD with other parasites and thus indicative of cotransmission events, and transmission intensity. The model-predicted proportion of within-host parasite diversity that is due to cotransmission events was shown to increase at lower transmission intensities (fig. 2*a*). We predict that at PCR prevalence <11.5%, >50% of strains within polygenomically infected individuals (COI>1) of all ages result from cotransmission events, rather than superinfection. This is based on the assumption that highly related parasites have originated from a recent common ancestor, and as such reflects the proportion of within-host genetic diversity that is due to cotransmission events rather than superinfection. We also predict this relationship is dependent on the age of individuals sampled, with parasites within younger individuals more likely to be more highly related. This reflects the increased chance that younger individuals will be treated after an initial infection due to their lower acquired immunity increasing the probability of developing clinical symptoms from an infection. Subsequently, younger individuals will be less able to accrue parasites from superinfection events, which increases the likelihood that any polygenomically infected individuals are the result of a cotransmission event. In figure 2*b*, the model-predicted relationship between mean IBD in mixed infections and the fraction of mixed infections is shown, and is well described by an exponential trend line fit to this data. The model-predicted relationship is comparable to estimates of IBD from whole-genome sequence data collected from sites across Africa and Asia as part of the Pf3k project (a collection of *P. falciparum* short-read sequences and associated analyses—https://www.malariagen.net/projects/pf3k) (Zhu et al. 2019). However, the model predicts significantly lower mean IBD in settings with a high fraction of mixed infections compared with the estimates based on the whole-genome sequencing data, with samples from sites in Ghana, Malawi, Mali, and the Democratic Republic of the Congo exhibiting higher mean IBD than predicted by the model.

### The Impact of Intervention Strategies on Parasite Genetic Diversity

**Fig. 3.**
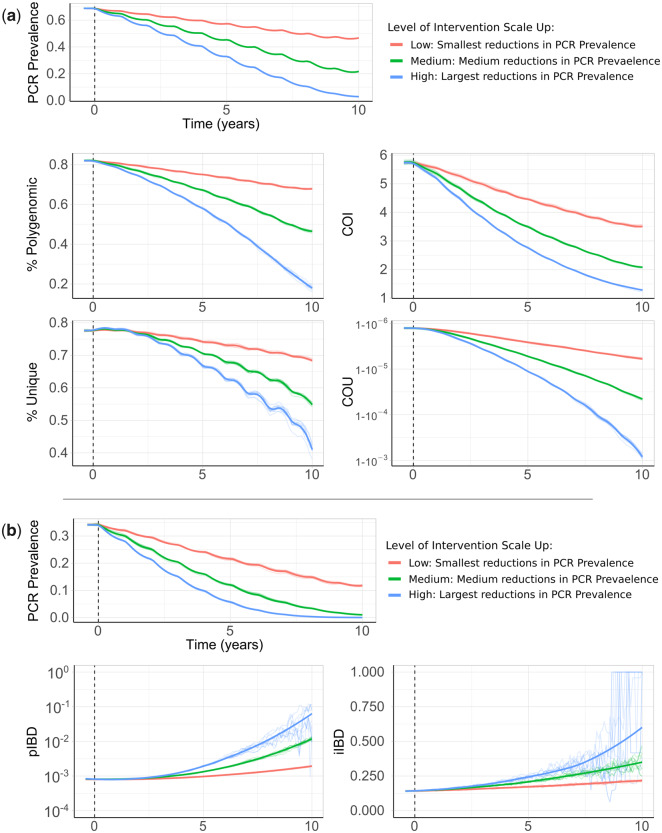
Impact of changes in transmission intensity upon genetic metrics of transmission intensity. In (*a*), the top plot shows the change in PCR prevalence after the introduction of three different levels of intervention scale-up, with both the ten individual stochastic realizations and the mean local regression smoothed relationship shown. The following four plots show the population mean percentage of the population that are polygenomically infected, the complexity of infection (COI), the percentage of samples that are genotypically unique (% unique) and the coefficient of uniqueness (COU) for the prevalence declines seen in the first row. COU measures the diversity of genetic barcodes, with COU=0 when all barcodes observed are identical and COU=1 when all barcodes observed are unique. In (*b*), the top plot shows the change in PCR prevalence, which starts at a lower starting prevalence of 35% compared with 70% in (*a*). The following row shows the within-host identity-by-descent (iIBD) mean across the 24 identity loci considered, and the population mean pairwise measure of IBD (pIBD). In all plots, the vertical dashed black line shows the time from which the scale-up of interventions starts (time=0 years).

Using our parameterized model, we first modeled how a reduction in transmission would affect four genetic metrics as the prevalence of malaria declined due to the scale-up of interventions (fig. 3). The genetic metrics explored were: (1) the population mean COI, (2) the percentage of samples that are polygenomic (COI>1), (3) the percentage of unique parasite 24-SNP barcodes, and (4) the coefficient of uniqueness (COU) (fig. 3). COU is a new measure of genetic relatedness within samples and is equal to 0 when all barcodes within a sample are identical, and is equal to 1 when all barcodes within a sample are unique (a multilocus analog of homozygosity).

The model was initiated at 70% PCR prevalence with no interventions in place. Three levels of intervention scale-up were simulated, representing a low, medium, and high reduction in prevalence resulting in a final PCR prevalence of ∼45%, ∼20%, and ∼5%, respectively, after 10 years. We predict that all four metrics decline proportionally with declining malaria prevalence (fig. 3*a*). The model predicts that the specific relationship depends on the population chosen for genetic testing (supplementary fig. 1*a*, Supplementary Material online). For example, COI is predicted to be higher in older age categories. The percentage of unique samples varied greatly depending on the subpopulation sampled, reflecting difference in the absolute numbers of individuals that fall within each subpopulation. Samples taken from individuals with asymptomatic infections were predicted to have the highest COI and percentage of polygenomic samples. Across the scenarios simulated, metrics based on the complexity of infection (COI and % Polygenomic) showed a higher level of correlation with changes in the prevalence of malaria than measures based on the uniqueness of samples (COU and % Unique) (table 2). In addition, samples collected only from patients with symptomatic malaria led to metrics that were the least correlated with reductions in prevalence, resulting from the decreased number of available samples. This effect was most noticeable when assessing the percentage of unique genotypes within clinical samples, which had a correlation coefficient of 0.24 with PCR prevalence (table 2).

**Table 2.** Kendall Rank Correlation Coefficients between Genetic Diversity Metrics and Parasite Prevalence.

Sampled	% Polygenomic	COI	% Unique	COU	iIBD	pIBD
All	0.97	0.96	0.83	0.93	−0.89	−0.86
0–5	0.96	0.96	0.73	0.93	−0.80	−0.86
5–15	0.97	0.96	0.83	0.93	−0.86	−0.86
15+	0.97	0.96	0.83	0.92	−0.84	−0.86
Clinical	0.87	0.91	0.24	0.75	−0.64	−0.85
Asymptomatic	0.97	0.96	0.83	0.93	−0.89	−0.86

Note.—Coefficients are bound between −1 and 1, with 1 indicating perfect-ranked positive correlation and −1 indicating perfect-ranked negative correlation.

We also assessed measures of parasite genetic diversity based on comparisons of the number of loci that are IBD, which included the within-host pairwise mean proportion of loci that are IBD (individual mean IBD [iIBD]) and the population pairwise mean proportion of loci that are IBD (population mean IBD [pIBD]). We predict that both metrics increase in response to declines in prevalence, however, we predict that pIBD only increases substantially at PCR prevalences <15% (fig. 3*b*). Consequently, metrics based on IBD were explored at a lower starting prevalence of 35% PCR prevalence before the scale-up of interventions. The shape of the increase in iIBD was predicted to be dependent on the population sampled (supplementary fig. 1*a*, Supplementary Material online), with iIBD increasing quicker in symptomatic individuals. iIBD, however, becomes less informative as transmission intensity declines, with individuals less likely to be infected with multiple strains due to the lower rates of superinfection.

### Power Analysis

**Fig. 4.**
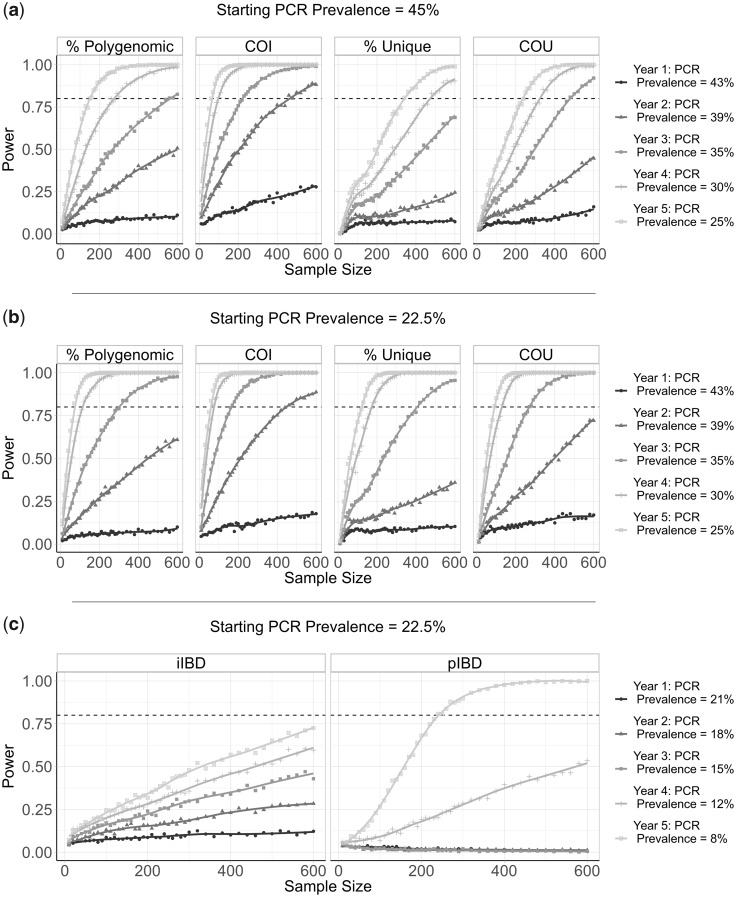
Predictive power of six metrics of parasite genetic diversity with respect to sample size. The distribution of sample means of six metrics of parasite genetic diversity was compared for 5 years following the initiation of the scale-up of intervention coverage. For each sample size, the power is defined as the proportion of 100 subsamples comparing year 0 and years 1–5 for which a significant difference in the mean was observed, estimated using one-tailed Monte Carlo *P* values generated by 100 permutations of the years samples were collected in. In (*a*), the metrics assessed are the percentage of samples that are polygenomic, the complexity of infection (COI), the percentage of barcodes within samples that are unique, and the coefficient of uniqueness (COU). The power of each metric was compared across 5 years in which a 20% absolute decrease in parasite prevalence from 45% was observed. The same information is shown in (*b*), but for a 14.5% absolute decrease in prevalence from 22.5% over 5 years. In (*c*), the metrics considered are the mean within-host identity-by-descent (iIBD) and the population mean pairwise measure of IBD (pIBD). In each plot, 80% power is shown with the horizontal dashed line.

To evaluate the performance of each metric for detecting annual changes in the prevalence of malaria, we calculated the statistical power for each metric at different sample sizes. In this analysis, we conducted analogous simulations as before but focusing on samples collected from children aged between 5 and 15 years old. We estimate that after 5 years of intervention scale-up, corresponding to an absolute decrease in malaria prevalence by PCR of 20%, no more than 350 samples are required for each metric explored (except for iIBD) to detect the change in transmission intensity 80% of the time (fig. 4). The predictive power, however, declined across all metrics when the effect size, that is, the decrease in prevalence, decreased. With 600 samples, each metric had <40% power to detect the decrease in prevalence after 1 year. The performance of each metric was additionally dependent on the starting prevalence, with metrics based on the uniqueness of samples (COU and % Unique) predicted to be more powerful at lower starting prevalences compared with higher prevalences (fig. 4*b*). Metrics based on measures of IBD were overall less powerful, with the predictive power of iIBD being <80% across all years and sample sizes (fig. 4*c*). pIBD only exhibited a predictive power >80% when detecting the largest change in prevalence between 22.5% and 8%, requiring over 225 samples.

The power of COU, % Unique, and pIBD were noticeably worse when it was assumed that samples from polygenomically infected individuals could not be phased (supplementary fig. 2, Supplementary Material online). Under this assumption, we assume that we are unable to observe the genotype of each strain and consequently only the major haplotype within an individual is available, that is, calling the most abundant allele at each locus of the barcode, which negates our ability to measure an individual’s iIBD. Across the full range of malaria prevalence simulated, measures of COI and COU were consistently predicted to be the most powerful, with % unique samples and IBD metrics demonstrating increased power to detect changes in transmission in areas with lower baseline transmission intensities where we predict the genetic variation to be lower.

### Statistical Model for Predicting Transmission Intensity

In order to translate the information, we have characterized into an effective tool for assisting surveillance programs, a statistical model was created to predict malaria prevalence using genetic metrics derived from parasite SNP genotyping. Due to the difficulty in phasing high-complexity infections, we assumed that all collected samples were unphased and as such we did not focus on metrics based on IBD when building our data set for training our statistical model.

**Fig. 5.**
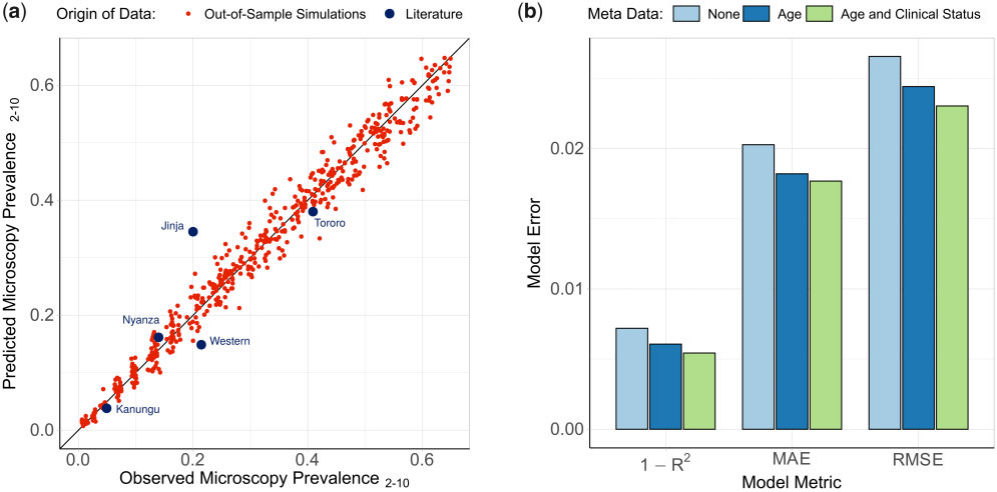
Ensemble statistical model-predicted malaria prevalence versus observed malaria prevalence. In (*a*), the performance of the trained ensemble statistical model is shown, with the model-predicted prevalence in red showing the predictions for the out-of-sample test data set composed of model simulations held back from model fitting. The blue points show the predicted prevalence for the five administrative regions considered earlier. In (*b*), the performance of the ensemble model is shown under different assumptions about the availability of patient metadata within simulated data.

The ensemble model was trained using the outputs of the developed transmission model, with simulations chosen that spanned the range of transmission, seasonality, and intervention coverage seen in sub-Saharan Africa. The resultant fitted models (combining three different statistical models: elastic net, gradient-boosted trees, and random forest) performed well on simulation data sets that were excluded from the model fitting, and was able to identify the underlying model behavior used to generate the training data set (fig. 5*a*). The best performing model provided accurate predictions of malaria prevalence when tested on SNP genotype data from the five administrative regions, with an observed mean absolute error (MAE) equal to 0.055 for these five locations. The performance of the model was enhanced when sample metadata was available (fig. 5*b*), with the ensemble model trained and tested using data with no age or clinical status information consistently performing worse. Similar patterns were also observed when assessing the performance of each of the level 1 models in the ensemble model (supplementary table 2, Supplementary Material online). As in the power analysis, across the range of malaria transmission intensities assessed, measures of COI and COU were observed to be the most informative metrics (supplementary fig. 3, Supplementary Material online). Model predictors based on the age and clinical status of individuals sampled contributed 28% toward the total model importance.

## Discussion

The substantial reduction in the cost of generating genetic data sets over the last 10 years and the establishment of scientific networks committed to generating and sharing genetic data has resulted in an abundance of sequenced *P. falciparum* genomes. This effort has resulted in the identification of loci associated with emerging drug resistance mechanisms (Cheeseman et al. 2012) and assisted in developing putative novel drug targets (Ludin et al. 2012). Another potential use of malaria sequencing efforts is understanding how malaria genomes can be used to study transmission. Simple population genetics principles predict that in a closed population a reduction in transmission intensity will typically be accompanied by a reduction in parasite genetic diversity, resulting from reduced opportunities for outcrossing to occur within the sexual stages of the parasite’s life cycle. However, there is as yet no consensus in the use of parasite genetics for inferring transmission intensity. There is a need to understand the contribution of superinfection and cotransmission toward the within-host parasite genetic diversity, which is often highlighted within critiques of early attempts to utilize modeling approaches for transmission intensity inference (Greenhouse and Smith 2015).

In this study, we have extended a previously developed model of malaria transmission to include individual mosquitoes and discrete parasite populations. The percentage of sporozoites that are successful within an infectious bite was estimated to be 20% (95% CI 10–29%), and was estimated by fitting our model to the COI and age of 3,002 individuals in five sites across Kenya and Uganda. The fitted model was used to initially estimate the proportion of the within-host parasite genetic diversity that is the result of cotransmission events resulting in the acquisition of highly identical parasite strains, as opposed to strains acquired through superinfection events. We predict that for malaria prevalence >11.5%, the majority of genetic variation within-hosts is generated through superinfection events. To our knowledge, this is the first attempt to characterize this relationship across the full transmission intensity spectrum seen within sub-Saharan Africa and represents a move toward standardizing which genetic metrics should be used at different transmission ranges. It is worth highlighting, however, that this finding is different to those of a recent study from Malawi, which observed a higher contribution from cotransmission events toward the genetic diversity of mixed infections (Nkhoma et al. 2020). One possible explanation is that in the Malawian study, samples were collected from young children, who were predicted to show higher levels of genetic relatedness within mixed infection (fig. 2*a*). This is because younger individuals have less immunity and are consequently more likely to develop clinical symptoms of malaria after an infection. This increases the chance that younger individuals will present at clinic after their first malaria infection, thus reducing the observed contribution of superinfections events toward the observed parasite genetic diversity within mixed infections.

We predict that IBD within samples decays exponentially as the proportion of samples is increasingly polygenomic. This exponential relationship was similar to findings in a recent study of IBD, which used whole-genome sequence data to explore this relationship (Zhu et al. 2019). However, the model predicted significantly lower IBD at higher transmission settings (settings with a higher fraction of mixed infections) than observed in the data presented in Zhu et al. (2019). There are a number of reasons for this. Firstly, the whole-genome sequence data were collected from individuals of unknown age as part of a convenience sample. If the samples were collected exclusively from younger individuals, the results in figure 2*a* would suggest that the mean IBD would be higher than if the samples were collected across all ages. Secondly, in the study by Zhu et al. (2019), the estimated COI across all sites was <2, which is significantly lower than COI estimates from the sites in Kenya and Uganda in figure 3*a*. Given that some of the African study sites in Zhu et al. (2019) are in areas of high transmission intensity, it seems likely that the convenience sampling scheme used has selected for individuals with lower COIs. One explanation could be that the individuals chosen for sequencing receive treatment more regularly, which reduces the probability of parasite strains from superinfection events being present at the time of sampling. This could be due to their age, or due to their enrollment in the study that resulted in them being selected for sequencing. Ultimately, without this information, it is challenging to draw strong conclusions about the validity of the model predictions in figure 3*b*, although the broad similarity is encouraging.

Our newly defined measure of parasite diversity, the COU, alongside COI were consistently powerful statistical tools for detecting changes in malaria prevalence. This is hardly surprising, as we should consider that the % unique samples and the % of polygenomic samples are simply the extreme cases of these metrics, and so we would expect them to contain less information. Additionally, the power analysis conducted was under the assumption that all samples that could be detected by PCR can be effectively phased. This is an overly ambitious assumption, and it is more correct to assess these metrics under the assumption that polygenomic samples cannot be phased (supplementary fig. 2, Supplementary Material online). However, the increase in statistical power when we are able to phase samples should highlight a need within the research field for methods to compare unphased parasite samples, with the majority of samples at higher transmission intensities predicted to have a COI >1.

In the absence of being able to phase polygenomic samples, however, the observed genetic metrics were still informative within the ensemble statistical model developed to translate parasite genetic information into estimates of malaria prevalence. For example, variable importance was observed for each predictor variable (supplementary fig. 3, Supplementary Material online), however, COU and COI accounted for nearly half the variance explained. There is also a degree of compensation afforded between metrics, that is, where one metric becomes less informative, another metric becomes more predictive. For example, at PCR PfPR (Plasmodium falciparum parasite rate) <10%, COI and the % of samples that are polygenomic will become substantially less informative, whereas IBD measures will start being more informative. This is further demonstrated by only needing 200 samples within our statistical ensemble model to produce accurate predictions of the prevalence of malaria, with the addition of individual-level metadata yielding further gains in model performance (fig. 5*b*). As more samples are added only modest improvements in model predictive performance are observed (supplementary fig. 4, Supplementary Material online). The importance of metadata, specifically the age of individuals, is highlighted in the findings of the model-predicted COI between age groups. In figure 3, we compared the COI between asymptomatic and symptomatic individuals, in which we predicted across all ages that asymptomatic individuals have higher COI. However, this finding does not hold when we compare the COI between symptomatic and asymptomatic individuals at different age groups and across different transmission intensities. For example, in the model fitting in lower transmission areas younger children who are symptomatic are predicted to have higher COI than asymptomatic younger children (supplementary fig. 5, Supplementary Material online). This finding is reversed, however, at higher transmission intensities reflecting the interaction between acquired clinical immunity and rates of superinfection. This pattern, however, may be different in other real-world settings, where other factors not modeled here, such as nonmalarial fevers and presumptive treatment may alter the effect that treatment has on the level of genetic diversity observed within individuals.

This study has some important limitations. Firstly, we assumed there is only one parameter detailing the percentage of sporozoites that successfully progress to a blood-stage, which is the same for all study sites considered. This is likely a simplification, but our observation of 20% sporozoites surviving from an individual mosquito feed is comparable to Bejon et al.’s (2005) observation of 25% (14 sporozoites surviving from an assumed total of 55 sporozoites resulting from five mosquito bites) of sporozoites successfully progressing to blood-stage infection. It is, however, higher than estimates based on transmission efficacy studies (Smith et al. 2010). The model fitting, however, revealed that the sensitivity to this parameter was low, with the confidence intervals for a value of *ζ* equal to 0.20 overlapping intervals for values of *ζ* ranging from 0.1 to 0.29. This is highlighted when we re-examined the model-predicted relationship between *msp2* COI and prevalence with these values, which showed only slight changes to the predicted COI (supplementary fig. 6, Supplementary Material online). However, It is important to highlight that this fitted value may not be representative of other African settings and that heterogeneity in transmission may result in a different percentage of sporozoites being successfully transmitted in other regions. The fitted estimate was also based on model fits to the administrative mean prevalence as opposed to the recorded prevalence in the specific study sites. For example, the study site in Jinja District, Walukuba, was observed to have the lowest parasite prevalence of all three study sites in Uganda (Nankabirwa et al. 2015). If we had used this prevalence value as opposed to the administrative prevalence value, the parameterized model would have failed to predict the pattern of COI in Walukuba (supplementary fig. 7, Supplementary Material online), which may suggest that this study site exhibits higher heterogeneity in the force of infection. However, the fact that the model-predicted COI closely matches the observed data when using the administrative region’s prevalence may suggest that parasite genetic metrics are more representative of the prevalence at larger spatial scales, which in turn may reflect human mobility between areas of differing transmission intensity and parasite genetic diversity. This may also be of benefit from a surveillance point of view, with 200 samples being able to give accurate measures of malaria prevalence within a large area. This could be of particular utility in areas where community surveillance is not feasible, in which samples collected from symptomatic patients attending public health facilities could provide additional information in helping to translate clinical incidence into measures of parasite prevalence.

Secondly, we did not explicitly model the scale-up of vector-based interventions, instead incorporating the effects of insecticide-treated nets (ITNs) and indoor residual spraying (IRS) through their impact on the average age of the mosquito population and the rate of anthrophagy. This assumption will cause each individual to experience the same relative reduction in molecular force of infection, that is, the number of new *P. falciparum* clones acquired over time. Consequently, model predictions are likely to underestimate the variance in the reduction of within-host parasite genetic diversity resulting from vector-based interventions. This effect would lead to a decrease in the statistical power of the genetic metrics considered and subsequently, the sample sizes presented within the power analysis are likely on the lower end of the sample sizes required for a given predictive power. Additionally, it is important to note that when estimating the statistical power of each genetic metric, these were conducted using model simulations conducted in nonseasonal settings. However, the bottlenecks resulting from seasonality in transmission are likely to have a large impact on the genetic diversity observed, in particular in low prevalence settings. This effect has been previously shown to lead to significant differences in estimates of *pfhrp2/*3 deletions in low transmission settings depending on the timing of sample collection within a transmission season (Watson et al. 2019). To circumvent this issue, parasite samples should be collected at the same point within a transmission season to increase the suitability of comparisons.

Thirdly, although the developed statistical model provided accurate estimates of malaria prevalence overall for the five regions, the prediction for Jinja was noticeably worse, which reflects the high COI observed in that region given its comparatively low prevalence. Although we were able to replicate the COI age relationship for this region during model parameterization, this was largely due to the fact that the historic prevalence for the region was much higher. For this reason, the model predicts that individuals in the region will have higher acquired immunity and will subsequently be able to harbor more infections before developing a fever and potentially being treated and thus clearing infections. The developed statistical model, however, did not include any covariates for historic prevalence or genetic diversity. Subsequently, predictions made by this model largely reflect the mean diversity expected for a given prevalence and will suffer when making predictions for regions that have experienced a recent and large decline in prevalence. Recent declines in prevalence will cause individuals in the region to possess higher immunity than predicted based solely on the region’s current prevalence, which has been shown to manifest in clear patterns in the size of the submicroscopic reservoir (Whittaker et al. 2019). From a genetic perspective, increased immunity may either lead to a reduction in within-host genetic diversity due to more infections being suppressed. Alternatively, increased immunity may increase within-host genetic diversity if the higher immunity decreases the frequency with which people develop clinical symptoms, which in turn reduces the likelihood that an individual has recently been treated and subsequently has cleared all parasite strains. The latter may be a possible explanation for the comparatively high COI observed in the Walukuba study site in Uganda compared with its malaria prevalence. Consequently, as more genetic data are collected over time, we will be able to extend the methods presented here to better handle recent changes in prevalence and incorporate historic measures of genetic diversity for more accurate predictions of malaria prevalence. Alternatively, the modeling framework presented here could be extended to incorporate alternative data sources, such as longitudinal measures of clinical incidence from passive surveillance.

In our model, we have only considered neutral genetic markers that are unlinked. Although these loci are informative for capturing standing genetic diversity, we have not considered how selective events may shape the genetic diversity. For example, if drug resistance was to spread quickly through an area it is likely that this would cause a decrease in genetic diversity in neighboring regions (ImWong et al. 2017). However, the precise impact that this will have on the metrics explored in this study will depend on both how quickly recombination will result in linkage disequilibrium decay and the strength of the selective sweep. Although these were not assessed in this article, it would be possible to adapt our model to consider loci under selection and simulate how known factors that affect the speed of selection, such as transmission intensity, importation of resistance, treatment rates, and the metabolic costs associated with resistance, impact genetic metrics. Lastly, the model could also be extended to better capture importation and spatial dynamics. The current model employs a continent-island assumption, where the genotypes of imported parasites are drawn from a population with a fixed population-level allele frequency. This could be extended to consider populations within a metapopulation, where importations are sampled from connected populations. This would have the benefit of better capturing dynamics between different populations and could incorporate different data sources such as mobile phone records and travel surveys, which have been used to give a greater resolution to the spatial dynamics of malaria transmission (Chang et al. 2019; Tessema et al. 2019).

The 2018 World Malaria Report shows that the reductions in the global burden of malaria made since 2000 may be stalling, with two million more cases of malaria estimated in 2017 compared with 2016 (World Health Organization 2018). These declines have necessitated the development of new tools to enhance current surveillance efforts. In this study, we have shown that that malaria genetic metrics could provide an additional toolkit for operational surveillance. In particular, a combination of metrics focused on the COIs, the frequency and uniqueness of genotyped barcodes, and measures of IBD could be used for inferring the prevalence of malaria across the current range of malaria prevalence. It is important to highlight that there is still a need to understand the cost-effectiveness of these tools compared with current surveillance methods. In many endemic areas, clinical incidence data provide a temporally and spatially rich measure of malaria transmission. However, it is reliant on the accuracy of estimates of the population size. In situations where this is not possible, such as migratory populations and clinics with unknown health facility catchment areas. Consequently, there may be a niche for parasite genetics to complement measures of malaria incidence in areas in which the spatial coverage of surveillance data is poor. It is hoped that these findings, in particular the importance of sample metadata and quantifying the contribution of cotransmission and superinfection events have in shaping genetic diversity, can guide future efforts by the wider community for utilizing malaria genotyping for epidemiological surveillance.

## Materials and Methods

### *Plasmodium falciparum* Transmission Model

An individual-level stochastic model was developed to simulate the transmission dynamics of *P. falciparum*. The model is based upon previous modeling efforts (Griffin et al. 2010, 2014, 2016; Watson et al. 2017), however with extensions to now include individual mosquitoes as well as humans, and with parasites now modeled as discrete populations associated with individual infection events. Each parasite population is identified by a 24-SNP barcode, with sexual stages represented by two barcodes to characterize the female and male gametes within the vector and allow recombination to be explicitly modeled. An overview of the original model is given here before describing the changes made to the model, with the full methods detailed in the supplementary methods, Supplementary Material online.

People exist in one of six infection states, with individuals beginning life susceptible to infection. At birth, individuals possess a level of maternal immunity that decays exponentially over the first 6 months. Each day individuals experience a force of infection that depends on their level of immunity, biting rate, and the abundance of infectious mosquitoes. Infected individuals, after a 12-day latent period, develop either clinical disease or asymptomatic infection dependent on their level of acquired immunity from previous infections. Individuals that develop disease have a fixed probability of being effectively treated. Treated individuals enter a protective state of prophylaxis, before returning to susceptible. Individuals that did not receive treatment recover to a state of asymptomatic infection. Asymptomatic individuals progress to a subpatent infection, before clearing infection and returning to susceptible. All infected individuals that are not in the prophylactic state are also susceptible to superinfection.

The adult stage of mosquito development is modeled individually, with adult mosquitoes beginning life susceptible to infection. Mosquitoes seek a blood meal on the same day they are born and every 3 days after that until they die. Infected mosquitoes pass through a latent infection stage that lasts 10 days before becoming onwardly infectious to humans. The introduction of vector-based interventions leads to a decrease in the average age of the mosquito population throughout the duration of the intervention due to the increased mortality rate. A decrease in anthrophagy is also observed reflecting mosquitoes that are repelled as a result of interventions but do not die. The daily rate of change to these parameters in response to ITN and IRS is calculated using an equivalent deterministic version of the earlier model that included interventions (Griffin et al. 2016), before being introduced as a time-dependent variable within the stochastic model.

### Parasite Genetics

Parasites are modeled as discrete populations that result from an infection event associated with a mosquito or a human (see supplementary methods, Supplementary Material online, for full description). Each asexual parasite is characterized by one genetic barcode, which contains information relating to 24-SNPs distributed across the parasite genome. In simulations modeling IBD, the barcode is modified to contain 24 integer values that uniquely index an individual in the starting population, enabling ancestry to be tracked over time and hence IBD rather than identity-by-state to be modeled. Sexual stages of the parasite lifecycle within the mosquito are represented by both a female and a male barcode, thus defining the range of recombinants that could be produced. During a successful human to mosquito infection event, multiple oocysts may develop within the mosquito. The number of oocysts formed is drawn from a zero-truncated negative binomial distribution with mean equal to 2.5 and shape equal to 1 (95% quantile: 1–9) (Churcher et al. 2013; Stone et al. 2013, 2014), with required gametocytes sampled from the human according to the relative parasitaemias of the gametocytogenic strains. This process results in more recently acquired parasite strains being more likely to be onwardly transmitted resulting from the assumed higher asexual parasite density. During a successful mosquito to human transmission event, multiple sporozoites may be onwardly transmitted, with the genotypes which are the result of recombination events from ruptured oocysts. Recombination is simulated at this stage, and generated recombinants stored within the mosquito and associated with the oocyst from which it originated. Within our simulations, we consider genetic loci that are unlinked. Consequently, the resultant sporozoites formed inherit each locus by sampling with equal probability from the parental genotypes. The number of sporozoites passed on is drawn from a zero-truncated geometric distribution with a mean of 10 (95% quantile: 1–29) (Beier et al. 1992; Bejon et al. 2005), with the percentage of sporozoites that survive estimated within model fitting.

### Model Fitting

Our extensions to the transmission model introduced a new parameter, *ζ*, which determines the percentage of the total sporozoites passed on within a feeding event that survive to yield a blood-stage infection and subsequently produce gametocytes. To fit this parameter, we compared the model-predicted relationship between the COI and age utilizing previously SNP-genotyped samples from five sites across Kenya (Omedo, Mogeni, Rockett, et al. 2017) and Uganda (Chang et al. 2017), collected between 2008–2010 and 2012–2013, respectively. In brief, dried blood spots were collected, and samples taken from individuals with evidence of asexual parasitaemia by microscopy were selected for Sequenom SNP genotyping. Genotyping was conducted using the Sequenom MassARRAY iPLEX platform, yielding minor and major allele frequencies.

We applied *THE REAL McCOIL* proportional method to the SNP-genotyped samples to estimate each individual’s COI (Chang et al. 2017). Samples were filtered first by excluding loci with >20% missing samples, followed by samples with >25% missing loci. We performed 30 MCMC repetitions for each sample, with a burn-in period of 10^4^ iterations followed by 10^6^ sampling iterations, with genotyping measurement error estimated along with COI and allele frequencies, and a maximum observable COI equal to 25. Default priors were assigned for each parameter, and we used standard methodology to confirm convergence between chains (Gelman and Rubin 1996).

The observed relationship between COI and age was compared with the model-predicted relationship for each administrative region studied. The model-predicted relationship was generated by conducting simulations calibrated to estimates of the administrative malaria prevalence from 2000 to 2015 (Bhatt et al. 2015), exploring 50 values of *ζ* between 0.5% and 50%. For each region, ten stochastic realizations of 100,000 individuals were simulated with a burn-in period of 50 years to ensure both an epidemiological and genetic equilibrium was reached by year 2000. In all simulations conducted in this study, the same population size and burn-in period were used throughout. For each of the five administrative regions of interest, we incorporate the historical scale-up of ITNs and IRS between 2000 and 2015, using data previously collated for the World Malaria Report (World Health Organization 2015), and estimates for the coverage of treatment modeled using DHS and MICS survey data (Cohen et al. 2012). Seasonality for each region was included by altering the total number of mosquitoes using annually fluctuating seasonal curves fitted to daily rainfall data from 2002 to 2009 (Cairns et al. 2012). Lastly, we introduced rates of importation of infections that were calculated for each year between 2000 and 2015 using a fitted gravity model of human mobility (Marshall et al. 2018). These sources represent infections acquired from individuals traveling out of the region and returning with an infection, and also mosquitoes being infected by individuals traveling from outside into to the region of interest.

We calculated the “distance” between our model predictions and the observed data using the Kullback–Leibler (KL) divergence (Burnham et al. 2002). Using an individual’s age and estimated COI, the distance between the observed and predicted distributions of COI for each age is given by:

$$I\left(\zeta_{i}\right)≔I\left(\mathrm{pCO}I_{i}\left(\xi \right),\mathrm{oCO}I_{i}\right)=\sum_{\mathrm{COI}=1}^{25}\mathrm{pCO}I_{i}\left(\xi \right)ln\left(\frac{\mathrm{pCO}I_{i}\left(\xi \right)}{\mathrm{oCO}I_{i}}\right),$$

where $\mathrm{oCO}I_{i}$ is the observed distribution of COI at age i and $\mathrm{pCO}I_{i}\left(\zeta \right)$ is one realization of the model-predicted distribution of COI at age i for a given frequency of successful sporozoites *ζ* (with only parasites that would have been detected by PCR being assumed to be detected by SNP genotyping). The total distance for a given value of *ζ* is subsequently given by:

$${\sum_{r}^{5}\left(\frac{\sum_{i}^{n_{i}}I\left(\zeta_{i}\right)w_{i}}{\sum_{i}^{n_{i}}w_{i}}\right)}_{r},$$

where $w_{i}$ is the weight for age i, and $n_{i}$ is the total number of unique sampled ages in administrative region r. This can be interpreted as the sum of the weighted KL divergence means within a region, with weights equal to the number of observations at each age. Each region thus contributes equally to the total distance, despite the difference in the number of individuals in each region.

Further model fit validation was conducted by incorporating a comparatively larger collection of estimates of the COI estimated using *msp2* genotyping, which is more commonly referred to as multiplicity of infection (MOI). *msp2* genotyping is known to underestimate COI in individuals with very high COIs, with COIs >7 difficult to observe. Consequently, to distinguish these estimates, we refer to these as *msp2* COI. We compiled *P. falciparum* malaria MOI data where there were estimates of both the malaria prevalence and the MOI of study participants. This was conducted by updating a previous review (Karl et al. 2016), using the same search terms of “falciparum multiplicity infection prevalence msp2.” Analogous relationships were predicted using the fitted model, with the model-predicted *msp2* COI estimated by assuming that any individual with a model-predicted COI >7 results in an *msp2* COI of 7, which reflects the limits of resolution when using *msp2* genotyping (Gupta et al. 2010).

### Contribution of Superinfection and Cotransmission Events toward Within-Host Genetic Diversity

The parameterized model was used to characterize the relative contribution of cotransmission events and superinfection events toward within-host parasite genetic diversity. Ten stochastic realizations of 100,000 individuals were simulated for 50 years at 15 different transmission intensities. The proportion of highly identical parasite strains (>50% of loci are IBD in pairwise comparison) within simulations was recorded and used to estimate the proportion of within-host genetic diversity that is due to cotransmission events rather than superinfection.

### Impact of Changes in Transmission Intensity upon Measures of Parasite Genetic Diversity

The effect of declines in transmission intensity on four measures of within-host genetic diversity was explored. The four measures considered were: (1) the mean COI, (2) the percentage of polygenomic infections (% Polygenomic), (3) the percentage of unique barcode genotypes (% Unique), and (4) a newly defined metric, the COU, which is given by:

$$\mathrm{COU}=1-\frac{(\sum_{i}^{n}x_{i}^{2})-\frac{1}{n}}{\left(1-\frac{1}{n}\right)};\;0\leq \mathrm{COU}\leq 1,$$

where $x_{i}$ is the frequency at which barcode i occurs within a sample of size n. COU=0 when all barcodes within a sample are identical, and COU=1 when all barcodes within a sample are unique.

Ten stochastic realizations of 100,000 individuals were simulated for 50 years with an initial parasite prevalence measured by PCR equal to ∼70% and a fixed importation rate to ensure both a genetic and an epidemiological equilibrium. Once at equilibrium, three differing levels of intervention scale-up (low, medium, high) were introduced that lead to an absolute reduction in parasite prevalence from 70% to 45%, 20%, and 5% after 10 years. The scale-up of interventions resulted in an increase in the coverage of ITNs (maximum after 10 years: 30%, 60%, and 90%), IRS (maximum after 10 years: 20%, 40%, and 60%), and treatment (maximum after 10 years: 15%, 30%, and 45%). For all simulations, the monthly mean for each genetic marker was recorded for the whole population as well as within three age ranges (0–5 years old, 5–15 years old, and over 15 years old), and within individuals who were asymptomatic or symptomatic at the time of sample collection.

An identical analysis was conducted at a lower starting prevalence, with maximum reductions in parasite prevalence by PCR from 35% to 20%, 2%, and ∼0% after 10 years, in order to assess the change in two measures of IBD, pIBD, and iIBD. The pIBD we define as the mean number of loci in pairwise comparisons between samples that are identical across all loci in terms of their 24-locus identity barcode (focusing on genotypes that could be detected by microscopy only), that is, it is the mean proportion of shared ancestry between samples. The iIBD is the mean number of identical loci of the 24-locus identity barcode within individuals who are polygenomically infected. If all sampled individuals are monogenomic, then iIBD is set equal to 1.

### Statistical Power Analysis of Parasite Genetic Measures

To evaluate the utility of the considered measures of parasite genetic diversity, we conducted an analysis to characterize the predictive power of each metric for detecting changes in transmission intensity, and their sensitivities to the sample size chosen. In an analogous design to earlier simulations, we conducted ten stochastic realizations of 100,000 individuals and measured sample mean measures of the COI, % Polygenomic, % Unique, COU, iIBD, and pIBD at yearly intervals for the first 5 years after the initiation of a 10-year scale-up of interventions.

Sensitivity to the sample size of each metric was assessed by sequentially sampling subsets of the simulated data and comparing the mean difference in metrics. Sample sizes between 10 and 600 individuals were explored, with 100 samples drawn from a stochastic realization at years 0, 1, 2, 3, 4, and 5, and comparisons made between years 1–5 and year 0, that is, 0–1, 0–2, … 0–5. All samples were collected from microscopy positive individuals aged between 5 and 15 years old. One-tailed Monte Carlo *P* values were generated for each subsample by 100 permutations of the years that samples were collected from. The power of each metric was defined as the proportion of subsamples for which 95% of the permuted mean differences were greater or less than the observed mean difference, with the direction of the tail dependent on whether the metric is expected to decrease or increase, respectively, in response to a decrease in transmission intensity. The overall power for each metric was calculated as the mean power of ten stochastic realizations, and repeated at two different starting parasite prevalence by PCR (∼45% and ∼22.5%). Metrics based on comparisons of IBD were only assessed for the lowest starting parasite prevalence. The performance of each metric was also explored under the assumption that it was not possible to phase all genotypes within the samples collected, and that only the dominant genotype was able to be called.

### Statistical Modeling of the Predictive Performance of Malaria Genetics for Surveillance

A statistical model was constructed to predict malaria prevalence using the genetic metrics explored thus far, with three different assumptions about the availability of patient metadata (no metadata, patient age only, and both patient age and symptomatic status of infection). Simulations of 100,000 individuals were conducted for 50 years for the purpose of constructing a simulated data set to be used to train the statistical model. Simulation settings were chosen to broadly reflect the epidemiology of malaria in sub-Saharan Africa, spanning across a wide range of transmission intensity (0–65% microscopy positive prevalence) (Weiss et al. 2019), seasonality (low and high seasonality with both unimodal and bimodal peaks in transmission explored) (Cairns et al. 2015), and intervention coverage (0–60% treatment, IRS, and ITN coverage) settings (Battle et al. 2016). To assess the operational utility of such a model for surveillance, samples of only 200 individuals were chosen from the simulations conducted at random from microscopy positive individuals of all ages. We used the sampled mean measures of the genetic metrics discussed, and, in models where patient metadata was assumed to be available, summaries of the age and clinical status of samples to create our simulated data sets. About 25% of the simulated data sets were held back as an out-of-sample data set to be used for evaluating the performance of the trained statistical models and to test for overfitting. Three different statistical models (gradient-boosted trees, elastic net regression model, and random forests) were fit to the model simulated data. The predictions of these level 1 models were subsequently used to train an ensemble model using a linear optimization based on the root mean-squared error (RMSE) of the level 1 models. When training both the level 1 models and the ensemble, K-fold cross-validation sets were produced by splitting the training the data into 25 sets of training data with the results of the cross-validation subsequently averaged to reduce any bias from the cross-validation set chosen. The averaged cross-validation results were used to assess the performance of the ensemble model on the testing data set by comparing the RMSE, MAE, and the correlation under the different assumptions about the availability of patient metadata. The predictors of the ensemble model were assessed for their contribution to the overall model performance. Variable importance was calculated for each level 1 model, before reporting their overall importance as the weighted mean importance, with the weight equal to the level 1 model weights in the ensemble model. Lastly, the trained ensemble model was used to predict the prevalence of malaria for the study sites considered within Uganda and Kenya.

## Supplementary Material

Supplementary data are available at *Molecular Biology and Evolution* online.

## Supplementary Material

msaa225_supplementary_data
