# Supplementary material for: Evaluating the Performance of Malaria Genetics for Inferring Changes in Transmission Intensity Using Transmission Modeling
Source: Mol Biol Evol. 2020 Sep 8;38(1):274–89. doi: 10.1093/molbev/msaa225 (PMC7783189; doi:10.1093/molbev/msaa225)
Supplement: msaa225_supplementary_data [file msaa225_supplementary_data.zip › Genomic_Utility_Supplementary Materials_MBE_revised_R2.docx]

**Evaluating the performance of malaria genetics for inferring changes in transmission intensity using transmission modelling**

**Supplementary Material**

**Supplementary Figure 1: Age and sampling dependent impact of changes in transmission intensity upon genetic metrics of transmission intensity.** In **a)** the top plot shows the change in PCR prevalence after the introduction of 3 different levels of intervention scale up, with both the 10 individual stochastic realisations and the mean local regression smoothed relationship shown. The following four rows show the population mean percentage of the population that are polygenomically infected, the complexity of infection (COI), the percentage of samples that are genotypically unique (% Unique) and the coefficient of uniqueness (COU) for the prevalence declines seen in the first row. The metrics are stratified into columns by the sampling scheme chosen. In **b)** the top plot shows the change in PCR prevalence, which reaches <1% in the highest intervention arm. The following rows show the within host identity-by-descent (iIBD) mean across the 24 identity loci considered, and the population mean pairwise measure of IBD (pIBD). In both the same sampling stratification is used as in **a).** In all plots the vertical dashed black line shows the time from which the scale up of interventions starts (Time = 0 years).


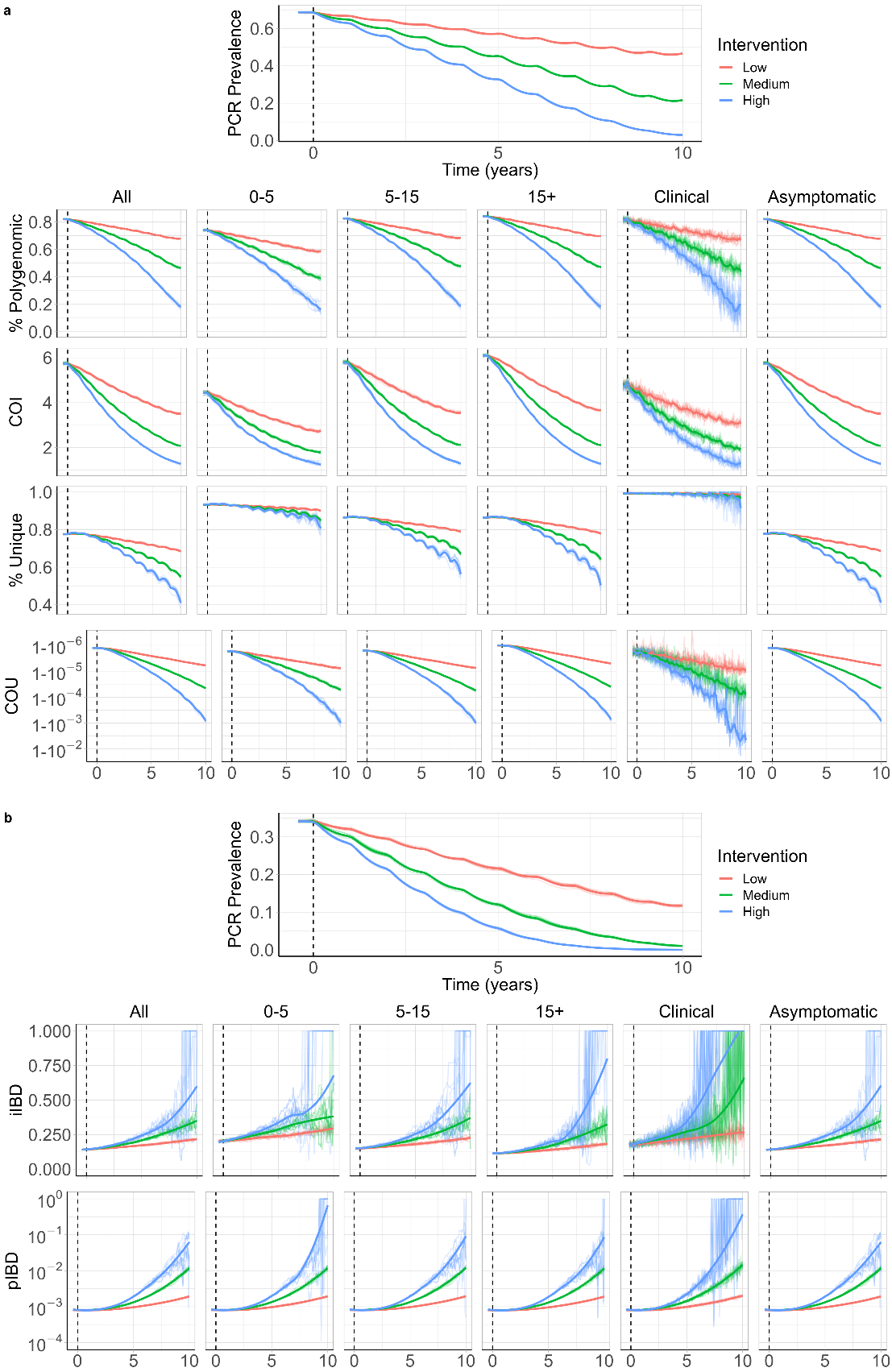


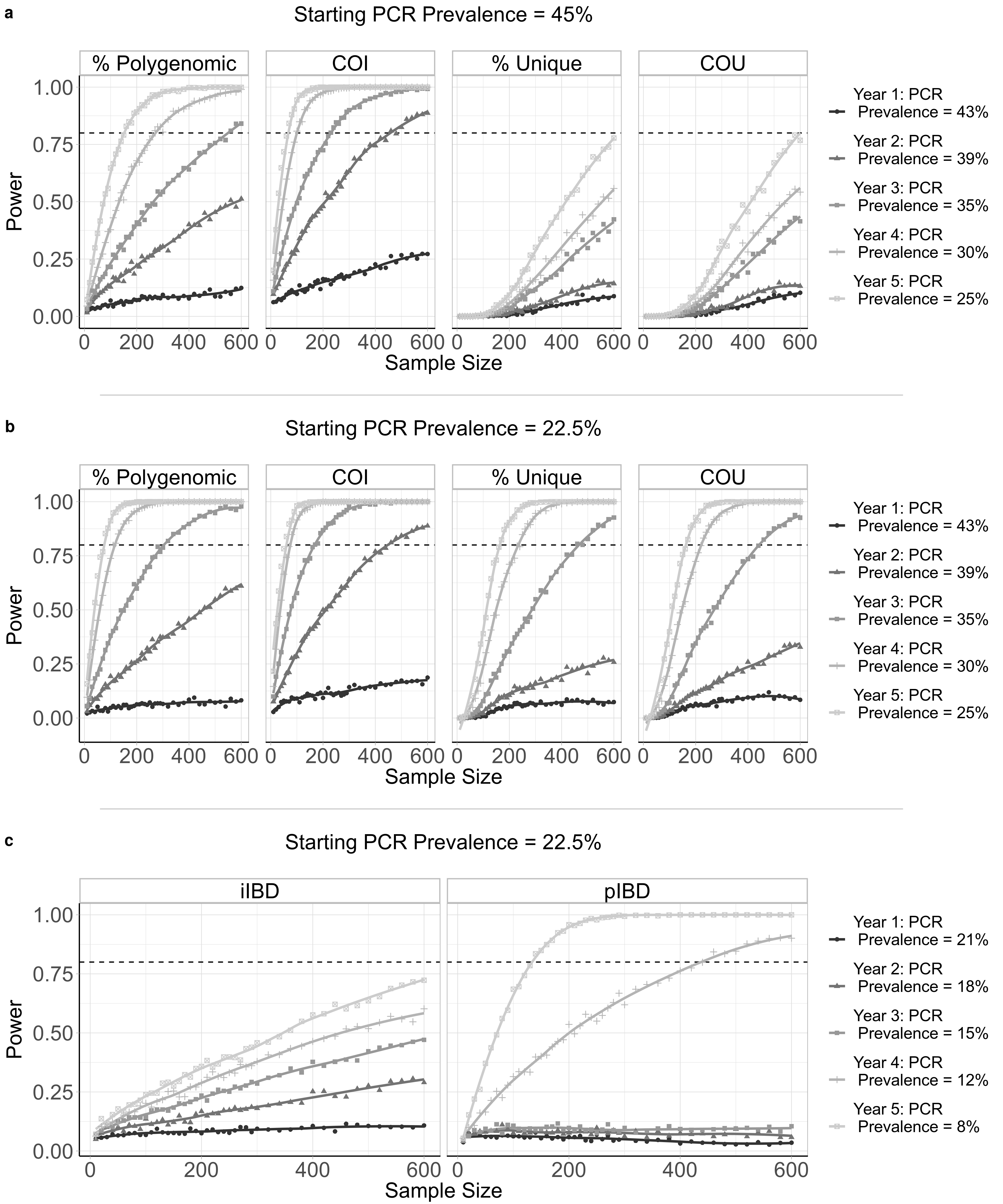


**Supplementary Figure 2: Predictive power of six metrics of parasite genetic diversity with respect to sample size under the assumptions that samples are unable to be phased.** The same methods as those detailed in the main text were used, with the only difference being that samples could not be phased and only the major haplotype could be called for an individual. iIBD is unable to be measured if samples cannot be phased and is subsequently crossed out. For pIBD, % Unique and COU it was assumed that the highest parasitaemia barcode was detected from each polygenomically infected individual. Lastly, there was no assumed difference in the ability to detect polygenomic samples or estimate the COI with unphased samples.


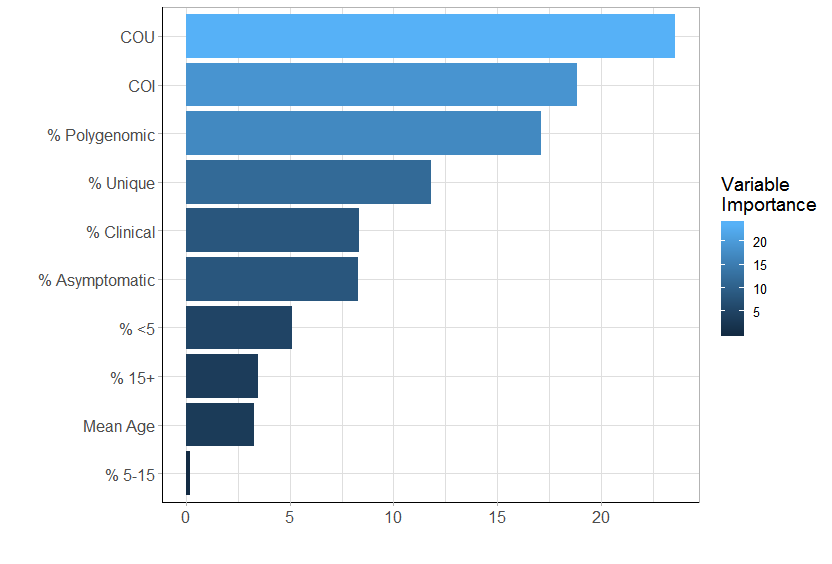
**Supplementary Figure 3: Mean Importance of each predictor variable within the trained ensemble model for predicting malarial prevalence.** The newly defined measure, the coefficient of uniqueness (COU), was observed to be the most important metric, with the six metadata variables (age and clinical status) being the least important. They do, however, contribute 28% of the total model importance, which highlights why the inclusion of this metadata resulted in better model predictions.


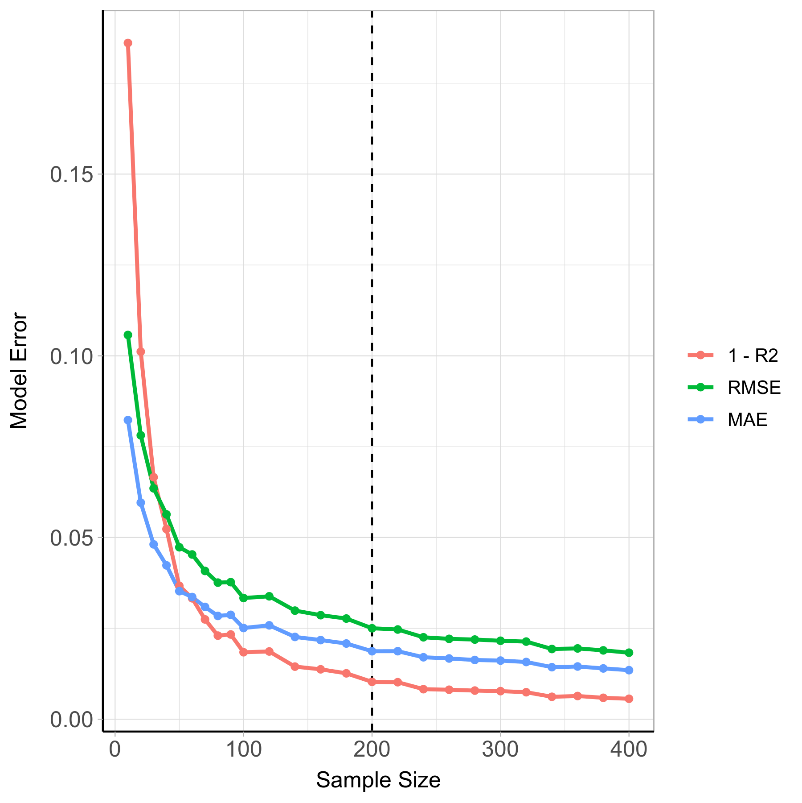


**Supplementary Figure 4: The predictive performance of the ensemble model under different assumed sample sizes.** Measures of the model error, root mean squared error (RMSE) and root mean error (MAE) as well as $1-R^{2}$ are shown for sample sizes between 10 and 400. Model performance improves quickly over sample size ranges between 10 and 100, before slowing, with only very modest increases seen in model performance for sample sizes larger than 200.


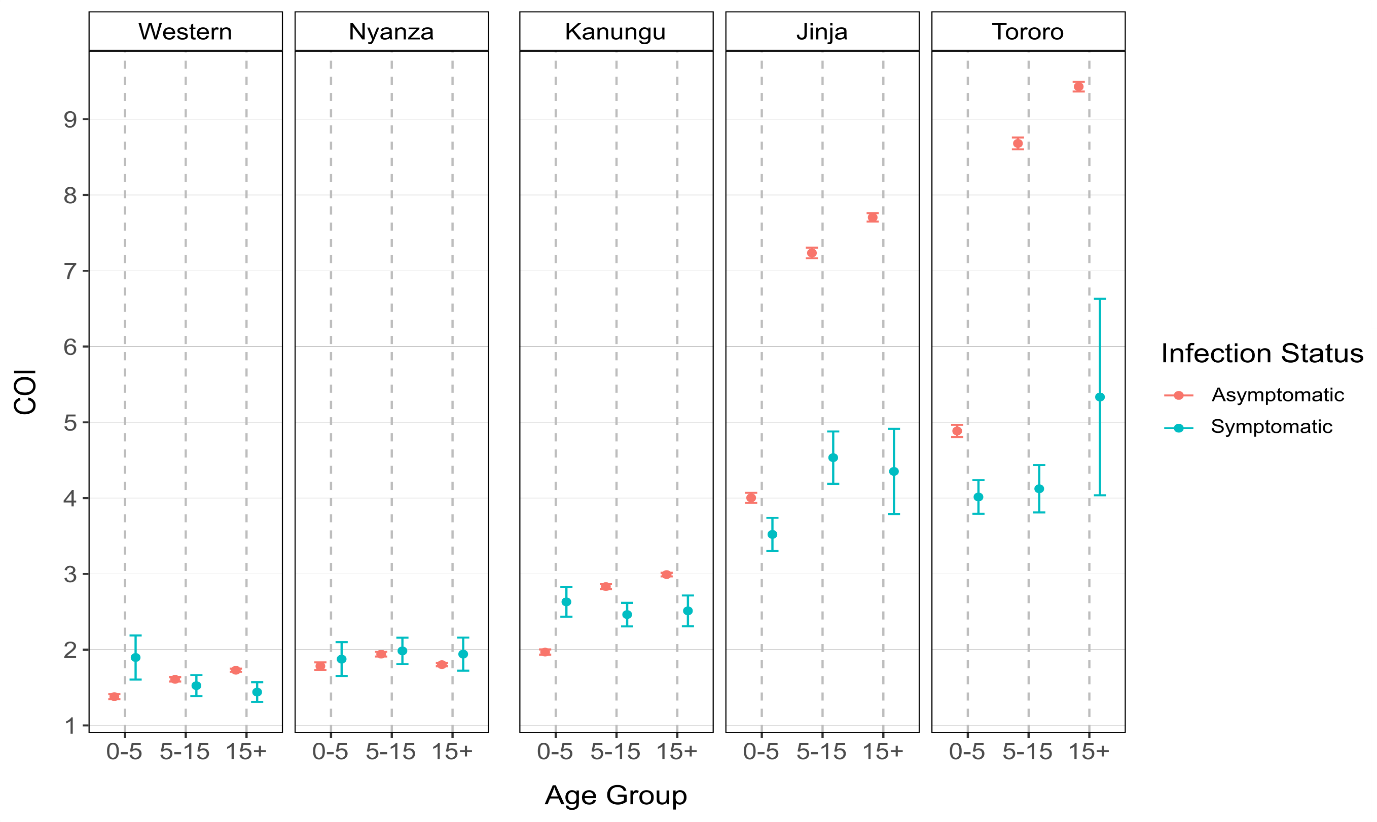


**Supplementary Figure 5: Age and symptomatic status stratified COI from model predictions during the model fitting.** Each plot shows the mean COI and 95% confidence interval for the study sites used in the model fitting. COI is stratified by age group and symptomatic status, showing that on the whole COI is higher in asymptomatic individuals, however, in lower transmission areas COI is higher in symptomatic young children.


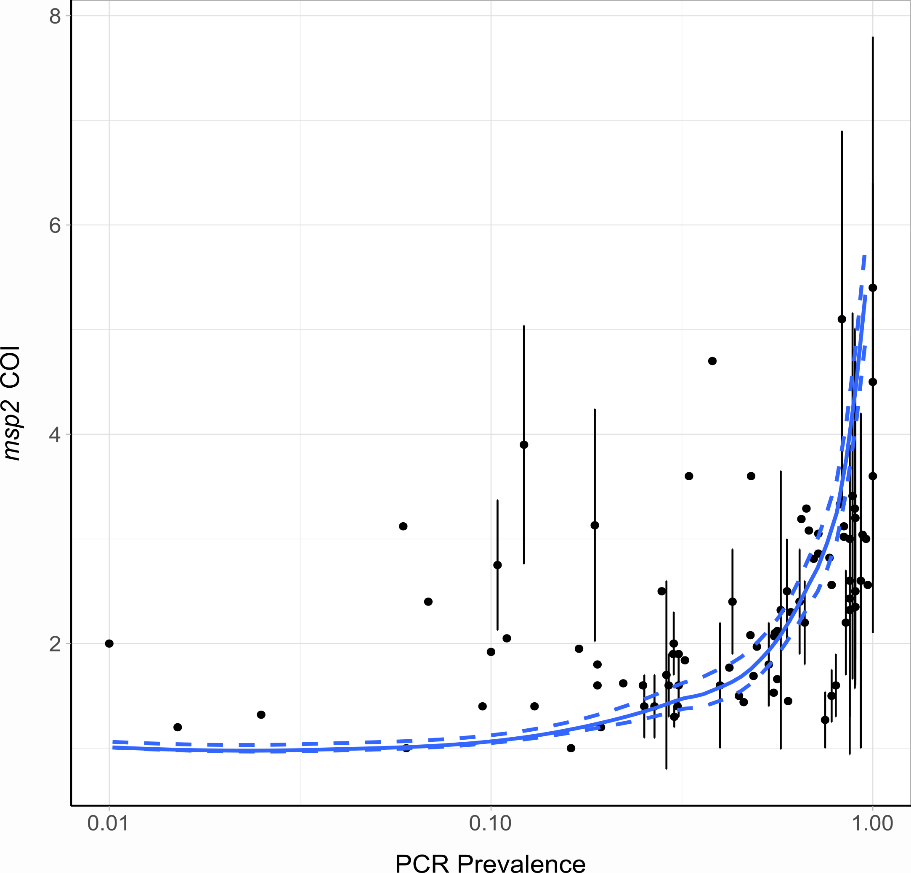


**Supplementary Figure 6: Model predicted relationship *msp2* COI and PCR prevalence.** The blue solid line shows the relationship for the fitted value of ζ equal to 0.20. The dashed lines above and below this in blue show the relationship for values of ζ equal to 0.29 and 0.10 respectively. The point-ranges in black show the observed values of COI by *msp2* genotyping from the literature review.

COI

Age

Age


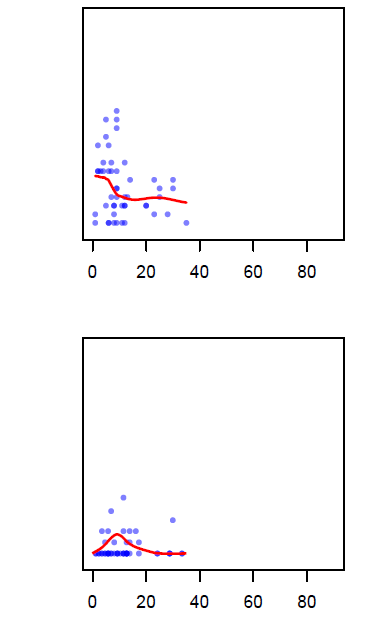

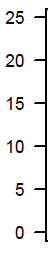

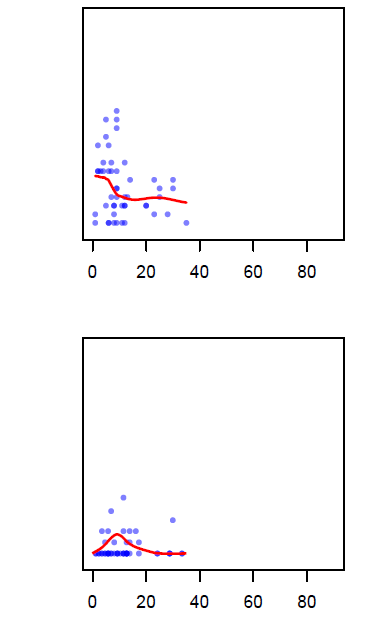

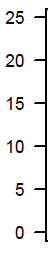


THE REAL McCOIL

MODEL OUTPUT

**Supplementary Figure 7: The fitted model-predicted relationship between COI and age for Walukuba, if the prevalence simulated was assumed to be equal to the prevalence within the sub-county surveyed, rather than the prevalence for the administrative region.** Model fitting conducted in Figure 1 in the main text used the administrative region prevalence as estimated by the Malaria Atlas Project, which resulted in good agreement between COI and prevalence.

**Supplementary Table 1: THE REAL McCOIL vs Model output estimates of mean COI by age**

See excel sheet - Supplementary Table 1 – for this table

| Supplementary Table 2: Statistical Model Performance. | | | | |
| --- | --- | --- | --- | --- |
| Meta Data | Model | RMSE* | MAE* | R^2^* |
| None | Elastic Net | 0.0276 (157.71%) | 0.0225 (173.08%) | 0.9935 (99.61%) |
| None | Gradient Boosted Trees | 0.0214 (122.29%) | 0.0159 (122.31%) | 0.9961 (99.87%) |
| None | Random Forest | 0.0211 (120.57%) | 0.0151 (116.15%) | 0.9962 (99.88%) |
| None | Weighted Mean Ensemble | 0.0204 (116.57%) | 0.0151 (116.15%) | 0.9965 (99.91%) |
| Age | Elastic Net | 0.0311 (177.71%) | 0.0245 (188.46%) | 0.9921 (99.47%) |
| Age | Gradient Boosted Trees | 0.02 (114.29%) | 0.0152 (116.92%) | 0.9967 (99.93%) |
| Age | Random Forest | 0.0197 (112.57%) | 0.0143 (110%) | 0.9968 (99.94%) |
| Age | Weighted Mean Ensemble | 0.0195 (111.43%) | 0.0144 (110.77%) | 0.9969 (99.95%) |
| Age and Clinical Status | Elastic Net | 0.0278 (158.86%) | 0.0219 (168.46%) | 0.9934 (99.6%) |
| Age and Clinical Status | Gradient Boosted Trees | 0.0178 (101.71%) | 0.0141 (108.46%) | 0.9974 (100%) |
| Age and Clinical Status | Random Forest | 0.0178 (101.71%) | 0.013 (100%) | 0.9973 (99.99%) |
| Age and Clinical Status | Weighted Mean Ensemble | 0.0175 (100%) | 0.013 (100%) | 0.9974 (100%) |
| * Absolute value (% relative to best performing model) | | |  |  |

# Supplementary Methods:

### *P. falciparum* Transmission Model

An individual-based stochastic model with a fixed daily time step was developed to simulate the transmission dynamics of *Plasmodium falciparum*. Both the human and adult mosquito stages are modelled at an individual level, whereas parasites are modelled as discrete populations with each population relating to an infection event. The human transmission model is based upon previous modelling efforts (Griffin et al. 2010; Griffin et al. 2014; Griffin et al. 2015; Griffin et al. 2016), which is described in its deterministic framework first, before detailing the human acquisition of immunity and the full set of equations detailing its stochastic implementation. The deterministic model described within the methods has been included as its equilibrium solution is used for model initialisation. Additionally, we developed a deterministic version of the earlier 2016 Griffin et al. model (Griffin et al. 2016) that incorporates interventions, which is used to indirectly incorporate the effects of intervention strategies as these are not modelled explicitly within the individual model. (The deterministic implementation of interventions has not been included within the deterministic model described below to ensure clarity related to our indirect handling of interventions).

We continue to describe the mosquito transmission model, which is again based on earlier modelling efforts (Griffin et al. 2010; Griffin et al. 2014; Griffin et al. 2015; Griffin et al. 2016), before describing the stochastic equations detailing the new implementation of the adult mosquito stage at an individual-based level. Extensions detailing how the parasite populations are incorporated follow, by first describing the genetic barcode that each parasite population possesses. We continue by describing the within host parasite populations, which includes considerations surrounding the contribution of coinfection and superinfection towards the model’s dynamics of within-host multiplicities of infection, and how these relate to the probabilistic uptake of specific gametocyte strains by mosquitoes. This is followed by detailing the within-mosquito parasite populations, which explores the derivation of the distribution describing the model-predicted oocyte intensities, and describes how recombination within the sexual stage is explicitly modelled.

#### Human transmission model

Individuals begin life susceptible to infection (state S) (Diagram 1). At birth, individuals possess a level of maternal immunity that decays exponentially over the first 6 months. Each day individual *i* is probabilistically exposed to infectious bites governed by their individual force of infection ($\Lambda_{i}$). $\Lambda_{i}$ is dependent on their pre-erythrocytic immunity, exposure to bites (dependent on both their age and their individual relative biting rate due to heterogeneous biting patterns by mosquitoes) and the size of the infectious mosquito population. Infected individuals, after a latent period of 12 days ($d_{E}$), develop either clinical disease (state D) or asymptomatic infection (state A). This outcome is determined by their probability of acquiring clinical disease ($\phi_{i}$), which is dependent on their clinical immunity. Individuals that develop disease have a fixed probability (*f_T_*) of seeking treatment (state T). Treated individuals are assumed to always recover, i.e. fully-curative treatment, and then enter a protective state of prophylaxis (state P) at rate *r_T,_* before returning to susceptible at rate$r_{s}$. Individuals that did not receive treatment recover to a state of asymptomatic infection at rate *r­_D_.* Asymptomatic individuals progress to a subpatent infection (stage U) at rate *r_A­_,* before clearing infection and returning to susceptible at rate *r_U_*. Additionally, superinfection is possible for all individuals in states D, A and U. Superinfected individuals who receive treatment will move to state T. Individuals who are superinfected but do not receive treatment in response to the superinfection will either develop clinical disease, thus moving to state D, or develop an asymptomatic infection and move to state A (except for individuals who were previously in state D, who will remain in state D).

**Diagram 1: Transmission Model.** Flow diagram for the human component of the transmission model, with dashed arrows indicating superinfection. S, susceptible; T, treated clinical disease; D, untreated clinical disease; P, prophylaxis; A, asymptomatic patent infection; U, asymptomatic sub-patent infection. All parameters are described and referenced within Table 1.

The movement between the human components of the transmission model is summarised with the following partial differential equations describing each compartment (*t* represents time and *a* represents age):

$$\frac{\partial S}{\partial t}+ \frac{\partial S}{\partial a}= -\Lambda(t-d_{E})S+\frac{P(t)}{d_{P}}+\frac{U(t)}{d_{U}}$$

$$\frac{\partial T}{\partial t}+ \frac{\partial T}{\partial a}= \phi f_{T}\Lambda(t-d_{E})(S(t)+D(t)+A(t)+U(t))-\frac{T(t)}{d_{T}}$$

$$\frac{\partial D}{\partial t}+ \frac{\partial D}{\partial a}= \phi{(1-f}_{T})\Lambda(t-d_{E})(S(t)+A(t)+U(t))-\frac{D(t)}{d_{D}}$$

$$\frac{\partial A}{\partial t}+ \frac{\partial A}{\partial a}=\left( 1-\phi\right)\Lambda(t-d_{E})\left( S(t)+U(t) \right)+\frac{D(t)}{d_{D}}-\phi\Lambda A(t)- \frac{A(t)}{d_{A}}$$

$$\frac{\partial U}{\partial t}+ \frac{\partial U}{\partial a}= \frac{A(t)}{d_{A}}-\frac{U(t)}{d_{U}}-\Lambda(t-d_{E})U(t)$$

$$\frac{\partial P}{\partial t}+ \frac{\partial P}{\partial a}= \frac{T(t)}{d_{T}}- \frac{P(t)}{d_{P}}$$

When an individual enters a new infection state a waiting time is sampled from an exponential distribution for when the individual will move out of that infection state (except when individuals move into S). With the introduction of a fixed daily time-step, the day on which an individual transitions from state X to Y occurs is given by:

$$Day\left( X \to Y \right) \sim floor\left( Exp\left( \lambda\right) \right)+t_{now}+1$$

where $t_{now}$ is the current day, i.e. the day that the individual moved into state A, and $\lambda$ is the transition rate. The set of state transitions for individuals and their associated transition rates are given below.

| Process | Transition | Transition Rate |
| --- | --- | --- |
| Progression of untreated disease to asymptomatic infection | D → A | $r_{D}=\frac{1}{d_{D}}$ |
| Progression of asymptomatic infection to subpatent infection | A → U | $r_{A}=\frac{1}{d_{A}}$ |
| Progression of subpatent infection to susceptible | U → S | $r_{U}=\frac{1}{d_{U}}$ |
| Progression of treated disease to uninfected prophylactic period | T → P | $r_{T}=\frac{1}{d_{T}}$ |
| Progression from uninfected prophylactic period to susceptible | P → S | $r_{P}=\frac{1}{d_{P}}$ |

We assume that each person has a unique biting rate, which is the product of their relative age dependent biting rate, $\psi_{i}$, given by

$$\psi_{i}\left( a \right)=\frac{\sum_{i=1}^{n} \psi_{i}\left( a \right)}{n}\left( 1-\rho\exp^{\frac{a}{a_{0}}} \right)$$

and an assumed heterogeneity in biting patterns of mosquitoes, *ζ_i_*, which we assume persists throughout their lifetime and is drawn from a log-normal distribution with a mean of 1,

$$\log\left( \zeta_{i} \right)\sim N\left( \frac{-\sigma^{2}}{2},\sigma^{2} \right)$$

where $1-\rho$is the relative biting rate at birth when compared to adults and $a_{0}$ represents the time-scale at which the biting rate increases with age. The product of these biting rates is subsequently used to calculate the proportion of the whole population’s bites that person *i* receives on a given day, $\pi_{i}$.

Their daily entomological inoculation rate (EIR),$\epsilon_{i}$, is thus calculated by multiplying by the number of infectious mosquitoes taking a blood meal from a human that day, which in turn yields their force of infection, which are given by:

$$\pi_{i}=\zeta_{i}\psi_{i}$$

$$\epsilon_{i}= I_{M\_Feeding}\pi_{i}$$

$$\Lambda_{i}= \epsilon_{i}b_{i}$$

where $I_{M\_Feeding}$is the size of the feeding infectious mosquito population, and $b_{i}$ is the probability of infection given an infectious mosquito bite.

The inclusion of individual mosquitoes results in the following stochastic implementation of infection. On any given day the number of infectious mosquitoes taking a blood meal from a human ($I_{M\_Feeding})$ will result in the same number of infectious bites. These bites are allocated by sampling from the multinomial distribution using the conditional binomial method (Davis 1993), where sample weights are equal to $\pi_{i}$. Upon receiving an infectious bite, an individual will move to an untracked infection state, *I*$,$which leads to either clinical disease (*D*), treated clinical disease (*T*) or asymptomatic infection (*A*). This leads to the following transition rates related to infection below.

| Process | Transition | Transition Rate |
| --- | --- | --- |
| Infection | S → I | $\Lambda_{i}(t-d_{E})$ |
| Super-infection from untreated clinical disease, asymptomatic infection or subpatent infection | D → I  A → I  U → I | $\Lambda_{i}(t-d_{E})$ |

The probabilities of progressing from state *I* to *D, T* or *U* are determined an individual’s probability of clinical disease, $\phi_{i}$, and the treatment coverage:

$$Prob\left( Clinical Disease \right)=\phi_{i}$$

$$Prob\left( Treated Clinical Disease | Clinical Disease \right)=f_{T}$$

The human population was assumed to have a maximum possible age of 100 years, with an average age of 21 years within the population yielding an approximately exponential age distribution typical of sub-Saharan countries. The day on which a human dies is thus allocated at birth by sampling from an exponential distribution with a mean equal to 21 years. When an individual dies, they are replaced with a new-born individual with the same individual biting rate due to heterogeneity in biting patterns.

#### Immunity and Detection Functions

We model 3 stages at which immunity may impact transmission, as in the existing Griffin et al model:

1. Pre-erythrocytic immunity,$I_{B}$; reduction in the probability of infection given an infectious mosquito bite.
2. Acquired and Maternal Clinical Immunity,$I_{CA}$ and$I_{CM}$ respectively; reduction in the probability of clinical disease given an infection due to the effects of blood stage immunity.
3. Detection immunity,$I_{D}$; reduction in the probability of detection and a reduction in the onward infectivity towards mosquitoes due to the effects of blood stage immunity.

Maternal clinical immunity is assumed to be at birth a proportion,$P_{M}$*,* of the acquired immunity of a 20 year-old and to decay at rate $\frac{1}{d_{M}} .$ The remaining three types of immunity are described by the following partial differential equations, which describe how immunity increases due to exposure from zero at birth and decreases over time:

$$\frac{\partial I_{B}}{\partial t}+ \frac{\partial I_{B}}{\partial a}= \frac{\epsilon}{{\epsilon u}_{B}+1}- \frac{I_{B}}{d_{B}}$$

$$\frac{\partial I_{CA}}{\partial t}+ \frac{\partial I_{CA}}{\partial a}= \frac{\Lambda}{{\Lambda u}_{C}+1}- \frac{I_{CA}}{d_{CA}}$$

$$\frac{\partial I_{D}}{\partial t}+ \frac{\partial I_{D}}{\partial a}= \frac{\Lambda}{{\Lambda u}_{D}+1}- \frac{I_{D}}{d_{ID}}$$

where each $u$ term represents the time during which immunity cannot be boosted further after a previous boost and each $d$ term represents the duration of immunity.

The probabilities of infection, detection and clinical disease are subsequently created by transforming each immunity function by Hill functions. An individual’s probability of infection, $b_{i},$ is given by

$$b_{i}=b_{0}\left( b_{1}+\frac{1-b_{1}}{1+\left( \frac{I_{B}}{I_{B0}} \right)^{\kappa_{B}}} \right)$$

where $b_{0}$ is the maximum probability due to no immunity, $b_{0}b_{1}$ is the minimum probability and $I_{B0}$and $\kappa_{B}$are scale and shape parameters respectively.

An individual’s probability of clinical disease, $\phi_{i}$, is given by

$$\phi_{i}=\phi_{0}\left( \phi_{1}+\frac{1-\phi_{1}}{1+\left( \frac{I_{CA}+I_{CM}}{I_{C0}} \right)^{\kappa_{C}}} \right)$$

where $\phi_{0}$ is the maximum probability due to no immunity, $\phi_{1}\phi_{0}$ is the minimum probability and $I_{C0}$and $\kappa_{C}$ are scale and shape parameters respectively.

An individual’s probability of being detected by microscopy when asymptomatic, $q_{i},$ is given by

$$q_{i}=d_{1}+\left( \frac{1-d_{1}}{1+\left( \frac{I_{D}}{I_{D0}} \right)^{\kappa_{D}}f_{D}} \right)$$

where $d_{1}$ is the minimum probability due to maximum immunity, and $I_{D0}$ and $\kappa_{D}$ are scale and shape parameters respectively. $f_{D}$ is dependent only on an individual’s age is given by

$$\frac{df_{D}}{da}=1-\frac{1-f_{D0}}{1+\left( \frac{a}{a_{D}} \right)^{\gamma_{D}}}$$

where $f_{D0}$ represents the time-scale at which immunity changes with age, and $a_{D}$ and $\gamma_{D}$ are scale and shape parameters respectively.

The probability that an infected individual infects a mosquito upon being bitten is proportional to both their infectious state and their probability of detection, with a lower probability of detection assumed to correlate with a lower parasite density. Individuals who are in state D (clinically diseased), state U (sub-patent infection) and state T (receiving treatment) contribute to an onward infection within a mosquito with probabilities$c_{D}$, $c_{U}$ *_­_*and$c_{T}$. In state *A*, contribution to an onward infection within a mosquito occurs with probability$c_{A},$ and is given by $c_{U}+\left( c_{D}-c_{U} \right)q^{\gamma_{I}}$ where $q$ is the probability of being detected by microscopy when asymptomatic, and $\gamma_{I}$ is a parameter that controls how quickly infectiousness falls within the asymptomatic state.

#### Human Stochastic Model Equations

Given the definitions above, the full stochastic individual-based human component of the model can be formally described by its Kolmogorov forward equations. As before, let $i$ index individuals in the population. Then the state of individual $i$ at time $t$ is given by$\left\{ j,k,t_{k},l,t_{l},m,t_{m},a,t \right\}$, where $a$ is age, $j$ represents infection status ($S,D,A,U, T$ or$P$), $k$ is the level of infection-blocking immunity and $t_{k}$ is the time at which infection blocking immunity was last boosted. Similarly, $l$ and $t_{l}$ denote the level and time of last boosting of clinical immunity, respectively, while $m$ and $t_{m}$ do likewise for parasite detection immunity. Let $\delta_{p,q}$ denote the Kronecker delta ($\delta_{p,q}=1$ if $p=q$ and 0 otherwise) and $\delta\left( x \right)$ denote the Dirac delta function. Defining $P_{i}$($j,k,t_{k},l,t_{l},m,t_{m},a,t$) as the probability density function for individual $i$ being in state $\left\{ j,k,t_{k},l,t_{l},m,t_{m},a,t \right\}$ at time $t$, the time evolution of the system is governed by the following forward equation:

$$\frac{\partial P_{i}\left( j,k,t_{k},l,t_{l},m,t_{m},a,t \right)}{\partial t}+\frac{\partial P_{i}\left( j,k,t_{k},l,t_{l},m,t_{m},a,t \right)}{\partial a}=$$

$$\delta_{j,S}\left[ r_{P}P_{i}\left( P,k,t_{k},l,t_{l},m,t_{m},a,t \right)+r_{U}P_{i}\left( U,k,t_{k},l,t_{l},m,t_{m},a,t \right) \right]$$

$$+\delta_{j,A}\left[ r_{D}P_{i}\left( D,k,t_{k},l,t_{l},m,t_{m},a,t \right) \right]$$

$$+\delta_{j,U}\left[ r_{A}P_{i}\left( A,k,t_{k},l,t_{l},m,t_{m},a,t \right) \right]$$

$$+\delta_{j,P}\left[ r_{T}P_{i}\left( T,k,t_{k},l,t_{l},m,t_{m},a,t \right) \right]$$

$${+\left( 1-b_{i} \right)\epsilon}_{i}\left( t-d_{E} \right)\left[ \delta_{j,S}+\delta_{j,D}+\delta_{j,A}+\delta_{j,U} \right]\mathcal{O}_{b}⋄P_{i}\left( j,k,t_{k},l,t_{l},m,t_{m},a,t \right)$$

$$+b_{i}\epsilon_{i}\left( t-d_{E} \right)\left[ \delta_{j,A}\left( 1-\phi_{i} \right)+\delta_{j,D}\phi_{i}\left( 1-f_{T} \right)+\delta_{j,T}\phi_{i}f_{T} \right]\mathcal{O}_{b}⋄\mathcal{O}_{c}⋄\mathcal{O}_{d}⋄\sum_{j^{'}\in\left\{ S,A,U \right\}} P_{i}\left( j^{'},k,t_{k},l,t_{l},m,t_{m},a,t \right)$$

$$+b_{i}h_{i}\left( t-d_{E} \right)\mathcal{O}_{b}⋄\mathcal{O}_{c}⋄\mathcal{O}_{d}⋄P_{i}\left( D,k,t_{k},l,t_{l},m,t_{m},a,t \right)$$

$$+\left[ r_{B}k\frac{\partial}{\partial k}+r_{CA}l\frac{\partial}{\partial l}+r_{ID}m\frac{\partial}{\partial m} \right]P_{i}\left( j,k,t_{k},l,t_{l},m,t_{m},a,t \right)$$

$$+\mu{\delta\left( a \right)\delta\left( t_{k}+T_{big} \right)\delta\left( t_{l}+T_{big} \right)\delta\left( t_{m}+T_{big} \right)\delta}_{j,S}\delta_{k,0}\delta_{l,0}\delta_{m,0}\sum_{j^{'}} P_{i}\left( j^{'},k,t_{k},l,t_{l},m,t_{m},a,t \right)$$

$$-\left[ {{\mu+r}_{P}\delta}_{j,P}+{r_{U}\delta}_{j,U}+{r_{D}\delta}_{j,D}+{r_{A}\delta}_{j,A}+{r_{T}\delta}_{j,P}+h_{i}\left( t-d_{E} \right)\left[ \delta_{j,S}+\delta_{j,D}+\delta_{j,A}+\delta_{j,U} \right] \right]P_{i}\left( j,k,t_{k},l,t_{l},m,t_{m},a,t \right)$$

Here$\mathcal{O}_{b}$, $\mathcal{O}_{c}$ and $\mathcal{O}_{d}$ are commutative integral operators with the following action on a density $\left( j,k,t_{k},l,t_{l},m,t_{m},a,t \right)$ :

$$\mathcal{O}_{b}⋄f= \delta\left( t-t_{k} \right) \int_{0}^{\infty} f\left( j,k-1,t-u_{B}-\tau,l,t_{l},m,t_{m},a,t \right)d\tau+ \theta\left( \frac{t-t_{k}}{u_{B}} \right)f\left( j,k,t_{k},l,t_{l},m,t_{m},a,t \right)$$

$$\mathcal{O}_{c}⋄f= \delta\left( t-t_{l} \right) \int_{0}^{\infty} f\left( j,k,t_{k},l-1,t-u_{C}-\tau,m,t_{m},a,t \right)d\tau+ \theta\left( \frac{t-t_{l}}{u_{C}} \right)f\left( j,k,t_{k},l,t_{l},m,t_{m},a,t \right)$$

$$\mathcal{O}_{d}⋄f= \delta\left( t-t_{m} \right) \int_{0}^{\infty} f\left( j,k,t_{k},l,t_{l},m-1,t-u_{D}-\tau,a,t \right)d\tau+ \theta\left( \frac{t-t_{m}}{u_{D}} \right)f\left( j,k,t_{k},l,t_{l},m,t_{m},a,t \right).$$

Finally, $\theta\left( x \right)$ is an indicator function such that $\theta\left( x \right)=1$ if $x<1$ and 0 otherwise.

For simulation, a discrete time approximation of this stochastic model was used, with a time-step of 1 day. For each individual$k$, $l$ and $m$ are set to zero at birth, while$t_{k}$, $t_{l}$ and $t_{m}$ are set to a large negative value $-T_{big}$ (to represent never having been exposed or infected, i.e. their immunity will always be boosted upon their first exposure or infection event). Each immunity term increases by 1 for an individual whenever that individual receives an infectious bite ($k$), or is infected ($l$ and$m)$, if the previous boost to$k$,$l$ and $m$ occurred more than$u_{B}$,$u_{C}$ and$u_{D}$ days earlier, respectively. Immunity levels decay exponentially at rate$r_{B}$,$r_{CA}$ and$r_{ID}$, where$r_{B}$,$r_{CA}$ and$r_{ID}$ are equal to$\frac{1}{d_{B}}$,$\frac{1}{d_{CA}}$ and $\frac{1}{d_{ID}}$ respectively.

#### Mosquito Population Dynamics

The adult stage of mosquito development was modelled individually and is similarly described in its deterministic framework before exploring its stochastic implementation. Adult mosquitoes will begin life susceptible to infection (*S_M_*), and will seek a blood meal on the same day they are born and every 3 days after that until the mosquito dies. Each feeding day, mosquito *i* will be exposed to a force of infection, $\Lambda_{Mi},$depending on the infection status and immunity of the human the mosquito is feeding on. The overall force of infection towards the mosquito population on a given day*,*$\Lambda_{M},$ is thus represented by the sum of the onward infection contributions from each infected human, delayed by$d_{g}$, delay due gametocytogenesis, which is given by

$$\Lambda_{M}=\alpha_{k}Q_{0}\left( \sum_{i=1}^{\Sigma_{D}} \pi_{i}c_{D}+\sum_{i=1}^{\Sigma_{T}} \pi_{i}c_{T}+\sum_{i=1}^{\Sigma_{A}} \pi_{i}c_{A}+\sum_{i=1}^{\Sigma_{U}} \pi_{i}c_{U} \right)\left( t-d_{g} \right)$$

where $\alpha_{k}$ is the daily rate at which a mosquito takes a blood meal, $Q_{0}$is the proportion of bites that are on humans (anthropophagy) and $d_{g}$represents the delay from emergence of asexual blood-stage parasites to sexual gametocytes that contribute towards onward infectivity. Infected mosquitoes then pass through a latent infection stage (*E_M_*) that will last 10 days representing the extrinsic incubation period for the parasite (*d_EM_*)$,$ before becoming infectious to humans (*I_M_*). Infectious mosquitoes remain infectious until they die. Whenever a mosquito dies, it is replaced with a new susceptible adult mosquito. Analogously to the human model, when a new adult mosquito emerges, the day on which it dies is drawn from an exponential distribution with a transition rate of $\mu_{M}$= 0.132 days. The differential equations summarising the adult stage of mosquitoes are given by

$$\frac{dS_{M}}{dt}={\mu_{M}M}_{v}-{\mu_{M}S}_{M}-\Lambda_{M}S_{M}$$

$$\frac{dE_{M}}{dt}=\Lambda_{M}S_{M}-{\mu_{M}E}_{M}-\Lambda_{M}\left( t-d_{EM} \right)S_{M}\left( t-d_{EM} \right){exp}^{-\mu_{M}d_{EM}}$$

$$\frac{dI_{M}}{dt}=\Lambda_{M}\left( t-d_{EM} \right)S_{M}\left( t-d_{EM} \right){exp}^{-\mu_{M}d_{EM}}-\mu_{M}I_{M}$$

where $\mu_{M}$ is the daily death rate of adult mosquitoes, and *M_v_* is the total mosquito population, i.e. *S_M_ + E_M_ + I_M_*.

#### Mosquito Stochastic Model Equations

As with the human transmission model, the full stochastic individual-based mosquito component of the model can be formally described by its Kolmogorov forward equations. As before, let $i$ denote each mosquito in the population, and *j* denote their infection status. Let $\delta_{p,q}$ denote the Kronecker delta function such that it equals 1 if $p=q$and 0 otherwise. Defining $P_{i}$($j,t$) as the probability density function for mosquito $i$ being in state $\left\{ j,t \right\}$ at time $t$, the time evolution of the system is governed by the following forward equation:

$$\frac{\partial P_{i}\left( j, t \right)}{\partial t}= \delta_{j,E_{M}}\left[ \Lambda_{Mi}\left( P_{i}\left( S_{M},t \right) \right) \right]+ \delta_{j,I_{M}}\left[ \Lambda_{Mi}\left( t-d_{EM} \right)\left( P_{i}\left( S_{M},t \right) \right) \right]+\delta_{j,S_{M}}\mu_{M}\left[ P_{i}\left( S_{M},t \right)+P_{i}\left( E_{M},t \right){+P}_{i}\left( I_{M},t \right) \right]- P_{i}\left( j,t \right)\left[ \mu_{M} +\Lambda_{Mi}\left[ \delta_{j,S_{M}} \right] +\Lambda_{Mi}\left( t-d_{EM} \right)\left[ \delta_{j,S_{M}} \right] \right]$$

#### Seasonality and Intervention Strategies

In simulations in which no seasonality is assumed, $M_{v}$remains constant throughout, i.e. whenever a mosquito dies it is always replaced. When seasonality is incorporated, the maximum value that $M_{v}$ can be oscillates with a period of 365 days. This corresponds to a change in the birth rate of mosquitoes that reflects an assumed impact upon the seasonal carrying capacityof the environment as a result of rainfall patterns upon mosquito larval stage development. In these simulations, when a mosquito dies, it will only be replaced if the current total number of mosquitoes is less than the maximum value that $M_{v}$ can be. In simulations designed to replicate regional settings, a rainfall curve, $R\left( t \right),$ was estimated from rainfall data from 2002 to 2009 for the related first-administrative unit using the first three frequencies of the Fourier-transformed data (Garske et al. 2013). The seasonal total mosquito population size, $M_{v}\left( t \right),$ is thus given by

$$M_{v}\left( t \right)={M_{v}}_{0}\frac{R\left( t \right)}{\bar{R}}$$

Where $\bar{R}$ is the mean annual rainfall, and ${M_{v}}_{0}$ represents the seasonal harmonic mean population size.

The computational constraints introduced by modelling individual mosquitoes and parasite population genetic dynamics necessitated modelling intervention strategies indirectly. This was handled by assuming that an introduction of intervention leads to a decrease in the average age of the mosquito population throughout the duration of the intervention due to an increased mortality rate. As a result, the average age reflects a new composite mortality rate due to both interventions and external causes. Similarly it leads to an increase in $Q_{0}$ to reflect mosquitoes that are repelled as a result of interventions but do not die. The daily rate of change to these parameters in response to ITN and IRS coverage is calculated using an equivalent deterministic version of the earlier model that included interventions (Griffin et al. 2016), before being introduced as a time-dependent variable within the stochastic model.

### Parasite Dynamics

#### Parasite Genetic Barcode

Parasites are modelled as discrete populations as a result of an infection event associated with a mosquito or a human. Each asexual parasite is characterised by one genetic barcode, which contains information relating to 24-SNPs distributed across the parasite genome. These SNPs represent an increasingly used general SNP-based molecular barcode that has been used for the identification and tracking of *P. falciparum* clones (Daniels et al. 2008). Sexual stages of the parasite lifecycle within the mosquito are represented by both a female and male barcode, thus defining the range of recombinants that could be produced. The within human parasite dynamics and model considerations are discussed first before exploring the within mosquito parasite life cycle and associated modelling implications. A schematic overview of the modelled parasite lifecycle stages is shown in Diagram 2.

In simulations modelling identity-by-descent (IBD), we extend the barcode to consider 24 “identity-loci”. An identity loci can take any integer value required, allowing true identities to be compared. In the SNP-loci barcode, each loci can only be 0 or 1, representing the minor and major allele for that barcode loci.

#### Within Human Parasite Dynamics

During a successful mosquito to human infection event, a number of asexual parasite barcodes are introduced into the human, which may be observed in the ensuing gametocyte genotypes when considering onward infectiousness from humans to mosquitoes. If the individual’s pre-erythrocytic immunity was boosted in the last $u_{B}$days no new parasite barcodes will be passed to the individual, otherwise more than one different asexual parasite barcode that will be observed in the ensuing gametocyte genotypes may be introduced during an infection event, representing cotransmission of genetically related parasites (if the mosquito was infected with more than one sporozoite genotype). The precise distribution describing the number of genotypes is unknown (Wong et al. 2017), but the mean number of sporozoites within an inoculation event is well characterised by a geometric distribution with mean equal to 10. The geometric mean will then be used to estimate the proportion of sporozoites that are successful, $\xi$, which yields the maximum number of successful sporozoites in an individual with no pre-erythrocytic immunity. If this number is less than 1, then a new total number of sporozoites is drawn until the maximum number of sporozoites after incorporating $\xi$ is greater than 0. The observed number of successful sporozoites is then calculated by conducting Bernoulli trials for all but one of the successful sporozoites (as we assume one has to survive to found the infection) to see if they are successful, calculated using the individual’s probability of infection, $b_{i}$. In summary this can be written as:

$$Total_{spz} \sim Geom\left( p_{spz} \right)$$

$$Max_{spz}=round\left( Total_{spz}.\xi\right)$$

$$Observed_{spz}=1+\sum_{1}^{Max_{spz}-1} bernoulli(b_{i})$$

There is no assumed maximum number of parasites, with individuals assumed to clear strains on the day that they would have moved from a subpatent infection to susceptible for the strain considered, i.e. each acquired strain follows an assumed trajectory in parasitaemia representative of a normal infection cycle, i.e. with a mean duration of infectiousness equal to $d_{A}+d_{U}$. Acquired strains can thus move “infection state” independently of the human’s infection state. For example, a given individual is infected on day 0 and develops an asymptomatic infection. The individual is scheduled to become subpatent on day 200, but they were bitten on day 150 and developed clinical symptoms and moved to state D. When this happens, the parasite density of the strain acquired on day 0 does not change and this strain will become a subpatent strain on day 200. After day 200, its probability of being onwardly transmitted is thus equal to $c_{U}.$ After the parasite has moved to become a subpatent strain, the day at which the strain would have been cleared, i.e. the individual would have moved from state U to S if they had not been superinfected, is drawn and assigned to the parasite. On this drawn day the subpatent parasite strain is assumed to have been cleared. By tracking parasites in this way we are able to track the relative parasitemias of each acquired strain, enabling more accurate sampling of within host parasite genetic diversity when passing on gametocytes to mosquitoes as well as enabling an equilibrium between clearing old strains and acquiring new strains, which represents the multiplicity of infection. This is shown in the schematic below (Diagram 2), which also details the key features of the barcode.

#### Within mosquito parasite dynamics

When a mosquito is infected, we sample from a zero-truncated negative binomial distribution that describes the distribution of oocysts that form from a feeding event. The choice of a zero truncated negative binomial represents the increasingly identified zero-inflated negative binomial that describes the relationship between oocyst prevalence and mean oocysts per mosquito in SMFA studies (Churcher et al. 2012; Stone et al. 2013; Stone et al. 2014). The related negative binomial distribution for the distribution of oocysts is given by

$$X_{oocysts} \sim NB(size_{oocysts}, {shape}_{oocysts})$$

where $X_{oocysts}$ represents the number of oocysts that will be formed, with mean equal to 2.5 and a shape equal to 1, which captures the mean and range of oocysts observed in natural *P. falciparum* infections (Churcher et al. 2013; Stone et al. 2013; Stone et al. 2014). For each oocyst formed, two barcodes are sampled from the infected host representing the female and male gametes that led to the oocysts formation. These two barcodes will result in up to 4 different potential genotypes (reflecting the immediate two step meiotic division that takes place after zygote formation) represented within the sporozoite population within the oocyst. When an infectious mosquito seeks a blood meal and leads to an onward infection, a value for $Observed_{spz}$ is sampled. The oocyst source for each onward infection within a coinfection is sampled from oocysts that have ruptured, i.e. the infection event that led to the oocyst occurred more than 10 days earlier. At this point recombination is simulated by randomly choosing either the male or female allele at each SNP position in the barcode. The random sampling in this represents the assumed independent segregation events resulting from the absence of genetic linkage between barcode SNP positions. Once a recombinant has been simulated it is stored and associated with the oocyst from which it came. If the same oocyst is chosen to lead to an additional infection, then the previously generated recombinant has a 25% chance of being onwardly transmitted and there is a 75% chance that a new recombinant is generated and subsequently saved. This process will continue in ensuing onward infection events that result from this oocyst until four recombinants have been simulated, at which point they each have a 25% chance of being onwardly transmitted. The above thus introduces an assumption that sporozoites will remain onwardly-transmissible for the remainder of the mosquito’s life, with no effect upon their relative probability of being onwardly transmitted in relation to sporozoites that resulted from a more recently ruptured oocyst.

####

**Diagram 2: Parasite Dynamics within the transmission model.** Individual mosquitoes are tracked, which allows for recombination to be modelled explicitly. Populations of parasite clones are tracked, and multiple oocysts are able to be formed from a feeding event, as well as multiple genetically distinct sporozoites onwardly transmitted. A "barcode" is associated with each parasite clone and can either represent biallelic SNPs, or unique identities that allow IBD to be calculated.

#### Importation Rate

The non-spatial, closed population nature of the model will result in the eventual fixation of a single genetic barcode. As such, when conducting simulations designed to replicate regional settings, an estimate of the importation rate was calculated, yielding to a daily probability that an infection is due to an imported case. The importation rate represents the sum of two different flows of infection into a regional setting:

1. Individuals who are infected outside the region while travelling to and from other areas
2. Visiting travellers from outside the region who infect mosquitoes within the admin unit

These two process are incorporated at the same stage within the model, whereby there is a temporally dependent daily probability that a generated recombinant genotype is due to an importation as follows

$$Prob\left( Importation \right)= \delta_{imports}(t)$$

where $\delta_{imports}(t)$ is the population proportion of new infections resulting from importations on a given day. This parameter changes over time to reflect changes in regional seasonality (both within the region and neighbouring regions), and different rates of change in malaria prevalence across neighbouring regions.(Cook et al. 2011) If the recombinant is due to an importation, then a random barcode is produced and passed on. This barcode will also be stored and associated with an oocyst within the mosquito considered if it was probabilistically determined to be due to the second flow of importation defined above (B), determined by the ratio of these two flows of infection. Predicted rates of the two flows of infection above are calculated for each year between 2000 and 2015 using a fitted gravity model of human mobility (Marshall et al. 2018).

### Model Parameter Values and Code Availability

All model parameters used are provided in Table 1. The transmission model was written in C++ and is available as the R package magenta ([www.github.com/OJWatson/magenta](http://www.github.com/OJWatson/magenta)). magenta v1.0.0 was used in the analysis presented (Watson et al.).

**Table 1:** Parameter estimates used within the model were taken from Griffin et al. 2014, (Griffin et al. 2014) 2015 (Griffin et al. 2015) and 2016 (Griffin et al. 2016)

| Parameter | Symbol | Estimate |
| --- | --- | --- |
| Human infection duration (days) | | |
| Latent period | $d_{E}$ | 12 |
| Patent infection | $d_{A}$ | 200 |
| Clinical disease (treated) | $d_{T}$ | 5 |
| Clinical disease (untreated) | $d_{D}$ | 5 |
| Sub-patent infection | $d_{U}$ | 110 |
| Prophylaxis following treatment | $d_{P}$ | 25 |
| Treatment and Importation Parameters | | |
| Probability of seeking treatment if clinically diseased | $f_{T}$ | Variable |
| Importation Rate | $\delta_{imports}$ | 0.01 |
| Infectiousness to mosquitoes | | |
| Lag from parasites to infectious gametocytes | $d_{g}$ | 12 days |
| Untreated disease | $c_{D}$ | 0.0680 day^-1^ |
| Treated disease | $c_{T}$ | 0.0219 day^-1^ |
| Sub-patent infection | $c_{U}$ | 0.000620 day^-1^ |
| Parameter for infectiousness of state A | $\gamma_{1}$ | 1.824 |
| Age and heterogeneity | | |
| Age-dependent biting parameter | $\rho$ | 0.85 |
| Age-dependent biting parameter | $a_{0}$ | 8 years |
| Daily mortality rate of humans | $\mu$ | 0.000180 |
| Variance of the log heterogeneity in biting rates | $\sigma^{2}$ | 1.67 |
| Immunity reducing probability of infection | | |
| Maximum probability due to no immunity | $b_{0}$ | 0.590 |
| Maximum relative reduction due to immunity | $b_{1}$ | 0.5 |
| Inverse of decay rate | $d_{B}$ | 10 years |
| Scale parameter | $I_{B0}$ | 43.879 |
| Shape parameter | $\kappa_{B}$ | 2.155 |
| Duration in which immunity is not boosted | $u_{B}$ | 7.199 |
| Immunity reducing probability of clinical disease | | |
| Maximum probability due to no immunity | $\phi_{0}$ | 0.791 |
| Maximum relative reduction due to immunity | $\phi_{1}$ | 0.000737 |
| Inverse of decay rate | $d_{CA}$ | 30 years |
| Scale parameter | $I_{C0}$ | 18.0237 |
| Shape parameter | $\kappa_{C}$ | 2.370 |
| Duration in which immunity is not boosted | $u_{C}$ | 6.0635 |
| New-born immunity relative to mother’s | $P_{M}$ | 0.774 |
| Inverse of decay rate of maternal immunity | $d_{M}$ | 67.695 |
| Immunity reducing probability of detection | | |
| Minimum probability due to maximum immunity | $d_{1}$ | 0.161 |
| Inverse of decay rate | $d_{ID}$ | 10 years |
| Scale parameter | $I_{D0}$ | 1.578 |
| Shape parameter | $\kappa_{D}$ | 0.477 |
| Duration in which immunity is not boosted | $u_{D}$ | 9.445 |
| Scale parameter relating age to immunity | $a_{D}$ | 21.9 years |
| Time-scale at which immunity changes with age | $f_{D0}$ | 0.00706 |
| Shape parameter relating age to immunity | $\gamma_{D}$ | 4.818 |
| Mosquito Population Model | | |
| Daily mortality of adults | $\mu_{M}$ | 0.132 |
| Daily biting rate | $\alpha_{k}$ | 0.333 |
| Anthropophagy | $Q_{0}$ | 0.92 |
| Extrinsic incubation period | $d_{EM}$ | 10 days |
| Negative Binomial shape parameter for distribution of oocyst frequencies upon infection | $shape_{oocysts}$ | 2.5 |
| Negative Binomial size parameter for distribution of oocyst frequencies upon infection | ${size}_{oocysts}$ | 1 |
| Human Parasite Parameters |  |  |
| Geometric distribution of total sporozoites in an infectious bite probability | $p_{spz}$ | 1/10 |
| Percentage of sporozoites successfully reaching blood-stage | $\xi$ | 20% (fitted) |

## References

Churcher TS, Blagborough AM, Delves M, Ramakrishnan C, Kapulu MC, Williams AR, Biswas S, Da DF, Cohuet A, Sinden RE. 2012. Measuring the blockade of malaria transmission - An analysis of the Standard Membrane Feeding Assay. Int. J. Parasitol. 42:1037–1044.

Churcher TS, Bousema T, Walker M, Drakeley C, Schneider P, Ouédraogo AL, Basáñez MG. 2013. Predicting mosquito infection from Plasmodium falciparum gametocyte density and estimating the reservoir of infection. Elife 2013:1–12.

Cook J, Kleinschmidt I, Schwabe C, Nseng G, Bousema T, Corran PH, Riley EM, Drakeley CJ. 2011. Serological markers suggest heterogeneity of effectiveness of malaria control interventions on Bioko Island, Equatorial Guinea. PLoS One 6:1–9.

Daniels R, Volkman SK, Milner DA, Mahesh N, Neafsey DE, Park DJ, Rosen D, Angelino E, Sabeti PC, Wirth DF, et al. 2008. A general SNP-based molecular barcode for Plasmodium falciparum identification and tracking. Malar. J. 7:223.

Davis CS. 1993. The computer generation of multinomial random variates. Comput. Stat. Data Anal. 16:205–217.

Garske T, Ferguson NM, Ghani AC. 2013. Estimating Air Temperature and Its Influence on Malaria Transmission across Africa. PLoS One 8.

Griffin JT, Bhatt S, Sinka ME, Gething PW, Lynch M, Patouillard E, Shutes E, Newman RD, Alonso P, Cibulskis RE, et al. 2016. Potential for reduction of burden and local elimination of malaria by reducing Plasmodium falciparum malaria transmission: a mathematical modelling study. Lancet Infect. Dis. 3099:1–8.

Griffin JT, Ferguson NM, Ghani AC. 2014. Estimates of the changing age-burden of Plasmodium falciparum malaria disease in sub-Saharan Africa. Nat. Commun. 5.

Griffin JT, Hollingsworth TD, Okell LC, Churcher TS, White M, Hinsley W, Bousema T, Drakeley CJ, Ferguson NM, Basanez MG, et al. 2010. Reducing Plasmodium falciparum Malaria Transmission in Africa: A Model-Based Evaluation of Intervention Strategies.Krishna S, editor. PLoS Med. 7:e1000324.

Griffin JT, Hollingsworth TD, Reyburn H, Drakeley CJ, Riley EM, Ghani AC. 2015. Gradual acquisition of immunity to severe malaria with increasing exposure. Proc. R. Soc. B Biol. Sci. 282:20142657.

Marshall JM, Wu SL, C HMS, Kiware SS, Ouédraogo AL, Touré MB, Sturrock HJ, Ghani AC. 2018. Mathematical models of human mobility of relevance to malaria transmission in Africa. Nat. Sci. Reports:1–27.

Stone WJR, Churcher TS, Graumans W, Van Gemert GJ, Vos MW, Lanke KHW, Van De Vegte-Bolmer MG, Siebelink-Stoter R, Dechering KJ, Vaughan AM, et al. 2014. A scalable assessment of Plasmodium falciparum transmission in the standard membrane-feeding assay, using transgenic parasites expressing green fluorescent protein-luciferase. J. Infect. Dis. 210:1456–1463.

Stone WJR, Eldering M, van Gemert G-J, Lanke KHW, Grignard L, van de Vegte-Bolmer MG, Siebelink-Stoter R, Graumans W, Roeffen WFG, Drakeley CJ, et al. 2013. The relevance and applicability of oocyst prevalence as a read-out for mosquito feeding assays. Sci. Rep. 3:3418.

Watson OJ, Verity, R, Hellewell J, Slater HC. magenta: Individual-based simulation model of malaria epidemiology and genomics. R package version 1.2.0. https://ojwatson.github.io/magenta/.

Wong W, Griggs AD, Daniels RF, Schaffner SF, Ndiaye D, Bei AK, Deme AB, MacInnis B, Volkman SK, Hartl DL, et al. 2017. Genetic relatedness analysis reveals the cotransmission of genetically related Plasmodium falciparum parasites in Thiès, Senegal. Genome Med. 9:5.
